# PD-1 Blockade Mitigates Surgery-Induced Immunosuppression and Increases the Efficacy of Photodynamic Therapy for Pleural Mesothelioma

**DOI:** 10.1158/2767-9764.CRC-24-0571

**Published:** 2025-05-23

**Authors:** Gwendolyn M. Cramer, Richard W. Davis, Emmanouil Papasavvas, Astero Klampatsa, Joann M. Miller, Shirron Carter, Ruth Ikpe, Min Yuan, Sandy Widura, R. Sonali Majumdar, Sally McNulty, Mary Putt, Andrew V. Kossenkov, Luis J. Montaner, Sunil Singhal, Edmund K. Moon, Steven M. Albelda, Keith A. Cengel, Theresa M. Busch

**Affiliations:** 1 Department of Radiation Oncology, University of Pennsylvania Perelman School of Medicine, Philadelphia, Pennsylvania.; 2 The Wistar Institute, Philadelphia, Pennsylvania.; 3 Department of Medicine, University of Pennsylvania, Philadelphia, Pennsylvania.; 4 Department of Biostatistics, University of Pennsylvania, Philadelphia, Pennsylvania.; 5 Division of Thoracic Surgery, Department of Surgery, University of Pennsylvania Perelman School of Medicine, Philadelphia, Pennsylvania.

## Abstract

Lung-sparing radical pleurectomy with intraoperative photodynamic therapy (PDT) demonstrates remarkable survival for patients with pleural mesothelioma. Nevertheless, most patients treated with this multimodal approach will develop local tumor recurrence. An understanding of potential causes of treatment failure is central to developing mitigation strategies. Surgery importantly reduces disease burden but also produces tumor-promoting inflammation, as demonstrated through transcriptomic analysis of pleural mesothelioma specimens. Using preclinical models in the setting of combination therapy, we separated the benefit of surgical resection from its counterproductive effects on therapeutic outcome. Specifically, we evaluated mechanisms by which surgically induced inflammation can be therapy-limiting in a murine model of tumor incision (TI) introduced by a surgical cut across the tumor. In this TI model, we identified distinct TI-altered patterns in innate and adaptive inflammatory cells in murine mesothelioma tumors, and we studied changes in these patterns with the addition of PDT. TI introduction of an immunosuppressive environment is established via upregulation of PD-1/PD-L1 expression on tumor cells, T cells, and myeloid cells that is partially resolved by PDT. Immune dysfunction is further mitigated by the addition of PD-1 blockade, leading to curative potential in a process that requires Ly6G^+^ neutrophils and CD8^+^ T cells. Overall, these studies suggest that, without PDT, surgical modulation of immune cell trafficking and functionality leads to systemic immunosuppression. This immunosuppressive state potentially interferes with the generation of antitumor immunity by PDT. However, targeted inhibition of surgery-induced signaling in the PD-l/PD-L1 pathway counteracts surgery’s immunosuppressive outcomes to enhance PDT efficacy in the intraoperative setting.

**Significance:**

Surgery combined with PDT extends survival for patients with mesothelioma, but these patients are still at risk for tumor recurrence, in part due to the immunosuppressive effects of surgery. We find, in a mouse model, that combining surgery, PDT, and immune checkpoint blockade maximizes the efficacy of these therapies.

## Introduction

The etiology of pleural mesothelioma (PM) commonly consists of asbestos embedment in the pleura of the lungs, triggering a chronic inflammatory response and mesothelial carcinogenesis. This process is supported by high levels of reactive oxygen species–producing phagocytic cells, inflammasome activation, and amplified growth factor signaling (1–3). Years after exposure, this can progress to PM, a relatively rare and highly aggressive cancer of the pleura. Histologic and biologic heterogeneities in PM within and between patients necessitate a treatment plan that is multidisciplinary and adaptive. Typically, this includes systemic therapy; in surgical candidates, definitive options include combinations of radiotherapy with cytoreduction through extrapleural pneumonectomy or extended pleurectomy/decortication (4). As local recurrence is common even with multimodal treatment, additional strategies to improve control of tumor could include fluorescence-guided resection to improve surgical efficacy (5), cytotoxic therapies such as hyperthermic intraoperative chemotherapy (6), and immunotherapies to boost immune response (7).

For patients with PM, surgical resection can reduce tumor burden to mitigate symptoms and facilitate adjuvant cytotoxic or immune-stimulating therapies. However, surgical debulking results in altered cytokine levels, such as decreases in IL-2 signaling and increases in IL-1/6/8/10 signaling, as well as the release of growth factors, clotting factors, and stress hormones (8) that lead to the expansion of regulatory and suppressive immune cells, including myeloid-derived suppressor cells (MDSC) and tumor-associated macrophages (TAM; refs. 9, 10). Although accumulation of these regulatory immune cells is essential in the context of wound healing, the resulting amplified expression of PD-1/CTLA-4 immune checkpoints, decreased T-cell proliferation, and impaired NK cell cytotoxicity result in an overall state of immunosuppression (11–13). This potentially contributes to tumor relapse. However, it may also be possible to reset the tumor microenvironment from a protumor to antitumor state by introducing adjuvant therapies during key windows of opportunity in the perioperative or postsurgical setting.

We are evaluating photodynamic therapy (PDT) as an adjuvant to surgery for PM in a randomized phase II trial of intraoperative PDT (NCT02153229) following encouraging results with earlier intraoperative PDT trials for PM (NCT01673074 and NCT02159742; ref. 14). PDT is a cytotoxic therapy that leads to immunogenic cell death using visible wavelengths of light to activate reactive oxygen species–producing photosensitizers localized within tumor cells (15). In addition to directly causing tumor cell death, PDT also releases large quantities of cytokines and cellular debris that are a source of tumor antigens, initiates an influx of neutrophils followed by other myeloid cells, and subsequently generates an adaptive immune response (16–18).

To determine how surgically induced immunosuppression in the tumor environment interacts with the immunogenicity generated by PDT, we utilized a previously validated mouse model comprising a tumor incision (TI) of mesothelioma flank tumors (19). In this model, tumors are incised to generate surgical inflammation without removal of any tissue; this importantly maintains equal tumor volumes at the time of PDT across experimental groups. Using the TI model, we found that PDT improved long-term control of tumor after TI, but PDT was unable to completely overcome the effects of immunosuppressive TI (20). These studies importantly found that TI produced a significant increase in splenic and intratumoral neutrophils/granulocytic MDSCs (G-MDSC) and a corresponding negative impact on CD8^+^ T-cell antitumor activity (20). Furthermore, although TI prior to PDT did not alter the direct cytotoxic effects of PDT, it did introduce transient hypoxia that can alter tumor cell and MDSC phenotypes through upregulation of immune checkpoints (21).

In this study, we assessed potential adjuvants to the combination of TI and PDT with the goal of fully restoring PDT’s antitumor immune effects. Immune checkpoint blockade (ICB) is particularly relevant toward this goal because surgery/TI can alter the tumor microenvironment to upregulate immune checkpoints and increase the suppressive activity of myeloid cells (9, 10, 12, 13). Blockade of checkpoints such as PD-1/PD-L1 leverages the capacity of T cells already present by removing barriers to their function. This is most effective in a tumor microenvironment with heightened immune checkpoint activity and in which an immunogenic treatment (such as PDT) has been initiated to stimulate T-cell infiltration and activation (22, 23). The objectives of this study were to ascertain the influence of surgical resection on local inflammatory changes in PM, evaluate the effects of TI and PDT on PD-1/PD-L1 expression in the tumor microenvironment, and determine the utility of PD-1 blockade to augment the antitumor efficacy of PDT in the setting of surgical immunosuppression.

## Materials and Methods

### Clinical trial

Patients with epithelioid PM were enrolled on a randomized phase II prospective trial of pleurectomy and postoperative chemotherapy with or without intraoperative PDT (NCT02153229). All subjects were treated in accordance with protocols approved by the Institutional Review Board at the Hospital of the University of Pennsylvania. As defined by protocol, all subjects received lung-sparing pleurectomy to achieve macroscopic complete resection (MCR) of PM burden. After the completion of MCR, half of the patients went on to receive light delivery for Photofrin-PDT as determined by the randomization scheme for this phase II trial (14). Surgeries were performed between June 2014 and June 2021.

### RNA sequencing

Multiple PM specimens were collected from each patient over the course of surgery, from initiation to MCR, and immediately flash-frozen in liquid nitrogen vapor. All tissues were then stored at −80°C until further processing. Supplementary Fig. S1A shows the RNA sequencing (RNA-seq) workflow from PM surgery through computational analysis. Patients were prioritized for RNA-seq analysis based on specimen availability at multiple time points throughout surgery and irrespective of treatment group (i.e., surgery/PDT vs. surgery alone) because all tumor tissues were collected prior to PDT. Specimen processing for RNA-seq was performed in bulk for 80 PM specimens from 33 unique patients. Frozen specimens were sliced into 20-μm-thick sections for a total of 20 to 30 mg tissue, and DNase I–treated total RNA was isolated using Zymo Research Direct-zol MiniPrep Kit with tissue homogenization (Qiagen) according to the manufacturer’s instructions. 3′ mRNA-seq libraries were generated from 100 ng of DNase I–treated total RNA using QuantSeq FWD Library Prep Kit (Lexogen) according to the manufacturer’s directions. Overall library size was determined using the Agilent TapeStation and the DNA 5000 ScreenTape (Agilent). Libraries were quantitated by RT-PCR (Kapa Biosystems). Libraries were pooled, and high-output, single-read, 75 bp next-generation sequencing was performed on a NextSeq 500 (Illumina).

Time data could not be immediately confirmed for some tumor specimens, so computational analysis was based on 72 specimens from 31 patients for determining the influence of surgery on the tumor transcriptome, and remaining specimens were later used for validation. The swimmer plot in Supplementary Fig. S1B indicates precisely when during surgery each analyzed PM specimen was collected for each patient. Additionally, patient characteristics are summarized in Table 1. STAR aligner (24) was used along with RSEM (25) to estimate gene expression using the hg39 version of the human genome with Ensemble transcriptome information. Raw counts were tested for significance of gene expression association with the time of collection using DESeq2 algorithm (26) with statistical model defined as “design = ∼ time”, i.e., gene expression was modeled against the time of sample collection over the course of surgery without taking into account individual patient effects to avoid overfitting, given variation in collection times across patients, some proximate time points, and patients with just one sample. DESeq2 normalized count values were used for heatmap expression visualization. An FDR of less than 5% was taken as a threshold for gene expression change significance. Significantly differentially expressed genes were then subjected to Qiagen’s Ingenuity Pathway Analysis (IPA; RRID: SCR_008653).

**Table 1:** Characteristics of patients (*n* = 31) whose PM specimens were used for RNA-seq analysis

Patient characteristic	Number of patients, *n* (%)
Sex	
Male	26 (84)
Female	5 (16)
Neoadjuvant therapy	
Yes	6 (19)
No	25 (81)
Final histology	
Epithelioid	27 (87)
Biphasic	4 (13)

Significant canonical pathways as identified by IPA at a FDR<5% significance threshold and predicted activation/inhibition *Z*-score of at least 2 were reported. Gene expression of significantly associated with time genes identified in the IL-6 and IL-1 pathways was visualized as expression derived from values normalized by DESeq2 counts, log_2_-scaled, and mean-centered. On indicated figures, reported correlations were performed using Spearman rank correlation. Additional information denoting samples from the same patient was also indicated. Estimation of immune cell infiltration from bulk RNA-seq data was performed using CIBERSORTx software (27). Estimated values were correlated with time and compared between patient groups or between times within the same patients.

In order to check that the generalized results obtained without taking patient information into consideration were not biased because of the effect of random patient information, expression changes of reported pathways within each patient were examined. Correlation of pathway expression changes between pairs of samples from the same patient versus difference in collection times between the samples was compared with correlation obtained using direct statistical model without including patient factor. RNA-seq raw datasets have been deposited in the Gene Expression Omnibus database (28) under accession number GSE272176.

### Cell culture

Murine mesothelioma cell lines AB12 (RRID: CVCL_4405), described as biphasic (29, 30), and AE17 transfected with ovalbumin (AE17O, RRID: CVCL_LJ85) with a more epithelioid morphology (31–33) were grown in DMEM supplemented with 10% FBS and 1% penicillin/streptomycin. Cell lines were cultured for a maximum of 10 passages for use in all animal studies before rethawing from frozen stocks. All cell lines were validated by IDEXX and periodically tested for *Mycoplasma* contamination. *Mycoplasma* testing was performed by IDEXX BioAnalytics using PCR evaluation for the detection of *Mycoplasma pulmonis* and *Mycoplasma sp* or by Cell Center Services, Department of Genetics, University of Pennsylvania, by Cambrex MycoAlert.

### Tumor propagation

Animal studies were approved by the University of Pennsylvania Institutional Animal Care and Use Committee within animal facilities accredited by the American Association for the Accreditation of Laboratory Animal Care. Tumors were propagated by subcutaneous injection of 1 × 10^6^ AB12 or AE17O cells into the right flank of 8- to 10-week-old female BALB/c mice (RRID: IMSR_CRL:028) or C57BL/6 mice (RRID: IMSR_CRL:027), respectively (Charles River Laboratories) and measured with calipers to calculate volumes according to the following formula: (tumor width)^2^ × (tumor length) × π/6. Tumors reached mean volumes of 100 mm^3^ after 6 days for AB12 and 8 days for AE17O, and treatment groups were randomly assigned at this point. Mice were euthanized for flank tumor growth after the tumor volume reached 400 mm^3^ or rarely for tumor ulceration or metastasis.

### TI

TI was performed as previously outlined (19). Mice were provided analgesia via subcutaneous injection of long-acting buprenorphine (ZooPharm) and anesthetized via inhalation of 1.5% to 1.75% isoflurane in medical air (VetEquip anesthesia machine). Anesthetic depth was monitored by toe pinch reflex. After depilatory-based hair removal, skin was cleaned via alternating betadine and ethanol. In a sterile field, an incision and skin flap were used to expose the tumor. The tumor was incised with a sterile scalpel blade to one-half its depth along the longest axis without any tissue removal, and skin incisions were closed via interrupted sutures. Mice were immediately provided subcutaneous saline and monitored daily.

### PDT

Mice were injected with 5 mg/kg Photofrin (Pinnacle Biologics) via the tail vein. One day later, mice were anesthetized by inhalation of isoflurane in medical air (VetEquip anesthesia machine), and 632 nm light was delivered through microlens-tipped fibers. Light was produced using a Ceralas Biolitec laser, measured using a Coherent LabMax TOP Laser Power Meter (Coherent), and delivered over a 1.1-cm spot at 75 mW/cm^2^ to a total fluence dose of 135 J/cm^2^. In treatment groups combining TI and PDT, PDT was performed 4 hours after TI.

### Cytokine profiling

The Mouse Cytokine Array (Proteome Profiler Array, R&D Systems, ARY006) was used to identify relative levels of specific mouse cytokines and chemokines in tumor tissues 24 hours after PDT. Tissues were lysed according to the manufacturer’s recommendations. Tumors were excised and minced in PBS with protease inhibitors (10 μg/mL aprotinin, Tocris # 4139; 10 μg/mL leupeptin, Tocris # 1167; and 10 μg/mL pepstatin, Tocris # 1190) and homogenized on ice, and triton X-100 was added to a final concentration of 1%. Protein quantification was performed via amido black protein assay. Equivalent amounts of protein from five tumors were combined for each condition for a total of 250 μg of protein per membrane. Membranes were imaged using an Odyssey imaging system (LICOR Biosciences). Pixel values for each spot were determined in FIJI, background signal from a negative region was subtracted, and duplicate spots for each cytokine were averaged. To quantify interleukin-1 receptor antagonist (IL-1Ra) in tumors, Mouse IL-1Ra/IL-1F3 Quantikine ELISA Kit (R&D Systems) was used according to the manufacturer’s recommendations. Lysates from tumors 24 hours after PDT were collected as described above. The total protein per sample was 4 μg per well for the IL-1Ra ELISA.

### Antibody depletion

*In vivo* depletion antibodies and isotype controls were purchased from Bio X Cell and diluted with saline as needed for intraperitoneal injections. For PD-1 blockade, 2.5 mg/kg αPD-1 (clone RMP1-14, #BE0146, RRID: AB_10949053) was injected 1 hour after PDT (or 5 hours after TI) followed by twice weekly injections for 3 weeks. Supplementary Fig. S8A–S8C shows the depletion of PD-1 in samples of untreated tumor control (TC) at 5 days compared with IgG2a isotype control. CD8 T cells were depleted with 1 mg/kg αCD8a (clone YTS 169.4, #BE0117, RRID: AB_10950145) injected 2 days before treatment, the day after treatment, and then twice weekly until tumors reached 400 mm^3^. Neutrophils were depleted with 350 μg αLy6G (clone 1A8, #BE0075-1, RRID: AB_1107721) 90 minutes after PDT, followed by a second dose after 4 days. Isotype controls were rat IgG2a (clone 2A3, #BE0089, RRID: AB_1107769) for PD-1 and Ly6G and rat IgG2b (clone LTF-2, #BE0090, RRID: AB_1107780) for CD8, with injections following the same schedule and dose as depletion antibodies.

### Flow cytometry

Tumors were analyzed by flow cytometry where indicated. Tumors were enzymatically digested and processed using a validated protocol (34). After enzymatic digestion, they were mechanically sheared on a 70-μm cell strainer using the plunger of a 5-mL syringe in the presence of R10 medium containing RPMI-1640, 10% heat-inactivated FBS, 1% penicillin/streptomycin, HEPES, and 55 μmol/L β-mercaptoethanol. The resulting pellets were washed with R10 and then resuspended in PBS and Live/Dead fixable fluorescent reactive dye (1:1,000, Invitrogen) for 15 minutes. After washing, samples were resuspended in flow buffer with mouse Fc block (1:100 in PBS, 2.4G2, BD Biosciences) for 10 minutes at 4°C, followed by cell-surface marker antibodies for 30 minutes at 4°C. Antibodies (and specific clones) included CD45 (30.F11, #103132, RRID: AB_893340), CD3 (17A2, #100204, RRID: AB_312661), CD8a (53-6.7, #100722, RRID: AB_312761), CD4 (GK1.5, #100414, RRID: AB_312699), CD49b (DX5, #108908, RRID: AB_313415), CD11b (M1/70, #101228, RRID: AB_893232), Ly6G (1A8. #127624, RRID: AB_10640819), Ly6C (HK1.4, #128018, RRID: AB_1732082), CD62L (MEL-14, #104435, RRID: AB_10900082), and PD-1 (RMP1-30, #109121, RRID: AB_2687080; all from BioLegend) and PD-L1 (B7-H1, #564715, RRID: AB_2687479; from BD Biosciences). The PD-1 RMP1-30 clone was chosen specifically for its ability to still detect PD-1 in the presence of blocking antibody clone RMP-14 (35). Beads (Invitrogen UltraComp eBeads Plus Compensation Beads) were used for compensation. Flow cytometric analysis was performed on BD FACSCanto II with FACSDiva Software (BD Biosciences) and analyzed using FlowJo 10.9 (RRID: SCR_008520) with positive gates for PD-L1 and PD-1 sets based on fluorescence minus one controls. All statistical analyses are performed on data from at least two independent experiments. Example gating strategies for tumor myeloid cells and T cells are shown in Supplementary Fig. S1.

### Proliferation assay

T cells from naïve mice were isolated from spleens using Miltenyi Pan T Cell Isolation Kit II and stained with 5 μmol/L CellTrace Violet (Thermo Fisher Scientific). Ly6G^+^ cells were isolated using Miltenyi Myeloid-Derived Suppressor Cell Isolation Kit from mouse spleens 2 days after treatment with indicated experimental conditions. Cells were plated at different ratios in triplicate in round-bottom 96-well plates from 1:4 to 1:8 T cell: myeloid cell ratios. Cells were cultured in RPMI-1640 (Gibco) with 10% FBS, 1% penicillin/streptomycin, 1% HEPES (Gibco 15630080), and 55 μmol/L β-mercaptoethanol (Millipore-Sigma, ES-007-E). CD3/CD28 Dynabeads (Invitrogen) were added to stimulate T-cell proliferation. The positive control was activated T cells alone. The negative control did not receive any activation beads. After 72 hours of coculture, Dynabeads were magnetically removed, and T cells were stained with Live/Dead fluorescent reactive dye (Invitrogen) and anti-CD8 and anti-CD4 antibodies (BioLegend). The percentages of dividing T cells identifiable through progressive loss of CellTrace Violet staining were compared among different conditions.

### Statistics

Data from flow cytometry, ELISA, and tumor response studies were plotted and analyzed in GraphPad Prism version 10 (RRID: SCR_002798). Comparisons between two groups were made using the Mann–Whitney nonparametric test. Comparisons among multiple groups for the T-cell proliferation assay were made using ordinary two-way ANOVA with the Tukey multiple comparisons test. Tumor responses were compared with time-to-event analysis of Kaplan–Meier plots using log-rank (Mantel–Cox) tests, taking into account censoring for animals that did not regrow their tumors to 400 mm^3^ by a prespecified time of 90 days. All mice that reached 90 days after treatment had a complete response (CR) with no active tumor growth. The type I error rate, unadjusted for multiple comparisons, was 0.05.

### Data availability

RNA-seq raw datasets have been deposited in the Gene Expression Omnibus database (28) under accession number GSE272176. Other data generated in this study are available upon request from the corresponding author.

## Results

### Pleurectomy for patients with PM upregulates IL-6 and IL-1 signaling

**Figure 1:**
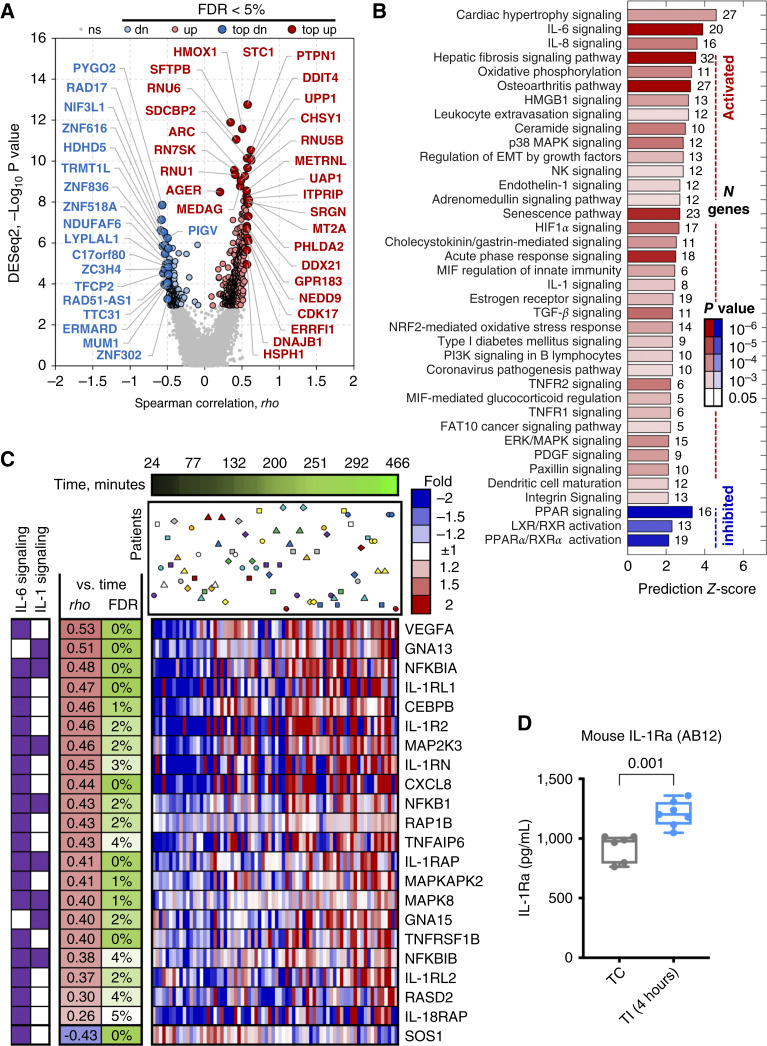
Mesothelioma surgery in humans and mice alters immune pathways. **A,** RNA-seq analysis was performed on tumor tissue isolated over the time course of surgery from 31 patients with PM. Volcano plot of significant gene expression changes associated with time of sample collection showing strength of correlation with time (Spearman rho) and significance of association based on DESeq2 algorithm. FDR <5% results are shown in red and blue with top genes by *P* value and/or correlation coefficients highlighted. dn, downregulated; ns, nonsignificant; up, upregulated. **B,** Analysis of FDR <5% genes significantly associated with the time of collection using IPA. Canonical pathways identified by IPA with FDR <5% and prediction *Z*-score >2 are shown. The color scale intensity, with red showing activated pathways and blue showing inhibited pathways, indicates significance of enrichment (*P* value). Numbers of differentially expressed genes in each pathway are indicated to the right of each bar. **C,** Heatmap showing log_2_ gene expression versus average across tumor specimens for members of the IL-6 and IL-1 families with specimens sorted by the time from initiation of surgery (green bar at the top). Specimens from the same individual patients (uniquely shaped and color-coded) are marked at the time of collection. IL-6 and IL-1 pathways both show increased activity as surgery progresses. **D,** In the AB12 mouse mesothelioma TI model, IL-1Ra protein levels are higher at 4 hours after TI (*n* = 7) compared with samples of untreated TC (*n* = 6, Mann–Whitney test *P* = 0.001).

Surgical resection can influence tissue behavior and immune interactions, leading to an altered inflammatory state. Our clinical trial delivers intraoperative PDT to the thoracic cavity after MCR of the tumor bulk. We have previously published evidence of systemic inflammation via increases in the circulating levels of IL-6 after MCR (19). Thus, we expect surgery to alter local signal transduction in the resection field by the time PDT is delivered. To detect these signaling changes, RNA-seq analysis was performed on tumor tissue isolated at different time intervals during the surgery from 31 patients with PM according to the experimental schema in Supplementary Fig. S1. Expression changes of 708 genes were found to be significantly associated with the time of sample collection (FDR <5%; Fig. 1A; Supplementary Table S1). Correlation analysis of changes in paired patient samples revealed that changes in gene expression are retained within each set of patient collection time points and are not a function of baseline patient variations (Supplementary Fig. S2A). Estimates of immune cell infiltrate via CIBERSORT demonstrated minimal changes over the time of surgery (Supplementary Fig. S2B).

Based on the IPA of differentially expressed genes, we discovered activity changes of several pathways significantly associated with tumor behavior were activated over the course of surgery (FDR <5%), including IL-6, IL-8, HMGB1, HIF1α, IL-1, TGFβ, and TNFα signaling (Fig. 1B). Activation of immune-specific pathways also included leukocyte extravasation signaling, NK cell signaling, MIF regulation of innate immunity, PI3K signaling in B lymphocytes, and dendritic cell maturation (Fig. 1B). Furthermore, PPAR activity was significantly inhibited as the surgical procedure progressed, which is linked to increases in TGFβ activity and other profibrotic cytokine signaling (36). Heatmaps of gene expression contributing to the activation of IL-6 and IL-1 pathways are shown in Fig. 1C. The IL-6 pathway was significantly affected (FDR<5%), with a predicted activation *Z*-score of 3.9 as time from the start of surgery approaches 8 hours (Fig. 1B). Similarly, the IL-1 pathway was significantly changed (FDR<5%) and predicted to be activated with time with a *Z*-score of 2.5 (Fig. 1B). Additional analyses did not support the association of changes in gene expression with the initial levels of gene expression, reported pathways, or immune cell sample composition as estimated by CIBERSORT.

In parallel with clinical studies, we demonstrated in the murine TI model of surgical inflammation that intratumoral IL-6 is significantly increased in AB12 mesothelioma flank tumors that undergo a surgical insult as compared with untreated control tumors (19, 20). Moreover, in this model, the levels of IL-1Ra (a member of both the IL-1 and IL-6 pathways) in the tumor increased (*P* = 0.001) at 4 hours after incision compared with untreated TCs (Fig. 1D). This is consistent with the increases detected in human PM specimens, in which IL-1RN gene expression is identified in the activated IL-6 pathway (Fig. 1C). Thus, using RNA-seq of PM tissue in patients undergoing MCR and recapitulated in murine models of TI by increases in IL-6 and IL-1Ra, these data suggest rapid increases in local inflammatory signaling in the surgical field immediately prior to PDT delivery.

### ICB modulates response after TI

Given these local increases in inflammatory signaling that accompany MCR and are modeled by TI, we hypothesized that the immune checkpoint axis of molecules may play a role in tumor responses to therapy after surgical inflammation. To assess whether immune checkpoints regulated tumor growth after surgical inflammation, we evaluated the effects of PD-1 ICB in mice with AB12 tumors that received TI, with key experiments repeated in AE17O tumors.

**Figure 2:**
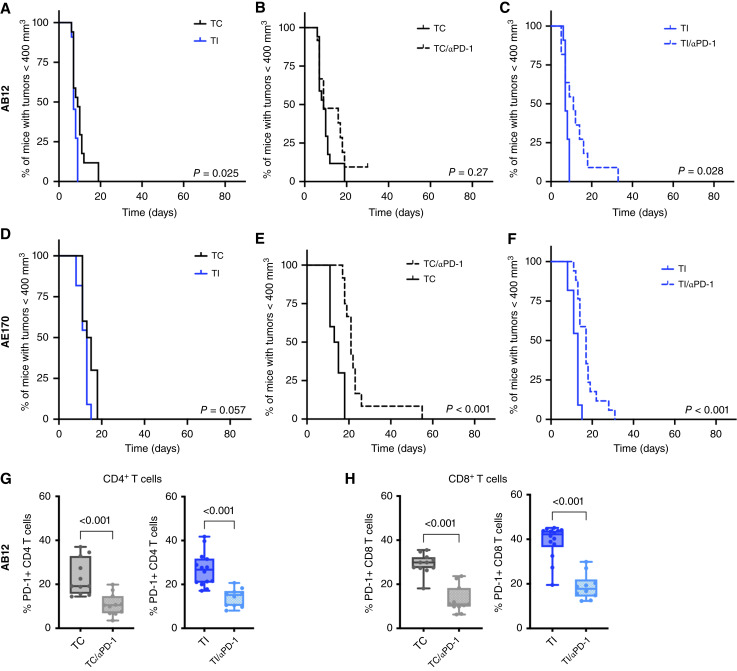
PD-1 blockade is minimally effective in AB12 and AE17O mouse mesothelioma tumors before or after TI. **A,** In subcutaneous AB12 mouse mesothelioma tumors, TI significantly decreased the median tumor growth time to 400 mm^3^ from 7 days compared with 9 days in untreated TC (*P* = 0.025). For TC, *n* = 17 mice and for TI, *n* = 11 mice. **B,** The addition of αPD-1 (TC/αPD-1, dotted black line) to TC did not significantly change tumor response (*P* = 0.27). In the TC/αPD-1 group, one mouse was censored because of tumor ulceration and subsequent early euthanasia. For TC/αPD-1, *n* = 12 mice. **C,** The addition of αPD-1 to TI led to significantly better outcomes with a slight delay in the median tumor growth time to 400 mm^3^ (7 days for TI and 11 days for TI/αPD-1; *P* = 0.028) For TI/αPD-1, *n* = 11 mice. **D,** In AE17O tumors, TI produced a modest insignificant increase in tumor growth compared with TC (*P* = 0.057 with a median of 13 days for TI vs. 14 days for TC). For TC, *n* = 10 mice and for TI, *n* = 11 mice. **E,** Adding αPD-1 in AE17O TC mice delayed the median time for tumor growth to 400 mm^3^ by 1 week from 14 days in TC mice to 21 days after TC/αPD-1 (*P* < 0.001). For TC/αPD-1, *n* = 12 mice. **F,** The addition of αPD-1 to TI also led to significantly better outcomes with a median time to 400 mm^3^ increasing from 13 days after TI to 17 days for TI/αPD-1 (*P* < 0.001). For TI/αPD-1, *n* = 12 mice. **G** and **H,** In AB12 tumors, flow cytometry of TC and TI tumors found a significant drop in PD-1 levels 2 days after a single αPD-1 dose for (**G**) CD4^+^ T cells and (**H**) CD8^+^ T cells. For TC *n* = 11 tumors, for TC/αPD-1 *n* = 11 tumors, for TI *n* = 14 tumors, and for TI/αPD-1 *n* = 10 tumors. Tumor responses (time to 400 mm^3^) are compared using a log-rank (Mantel–Cox) test; comparisons of continuous outcomes used a Mann–Whitney test.

The introduction of TI and its associated inflammation led to modest but significantly faster tumor growth, measured as the time to reach the flank tumor experimental endpoint of 400 mm^3^, compared with untreated TCs (*P* = 0.025 for TI vs. TC in AB12, Fig. 2A; *P* = 0.057 in AE17O; Fig. 2D). TI acts in this instance to model the inflammation of surgery—it does not involve the removal of tumor tissue and thus does not introduce the benefit of surgical resection. Consequently, TI-associated increases in tumor growth rates versus TC suggest a role for surgically induced inflammation in promoting tumor growth. When ICB was added as αPD-1 (Fig. 2B and E), the growth rate of TC versus TC/αPD-1 was unchanged in AB12 tumors and slightly slowed by αPD-1 in AE17O tumors (*P* < 0.001). However, αPD-1 initiated after TI led to small but significant slowing of tumor growth in both tumor models. Although all mice experienced tumor recurrence, we detected an increase in median time to 400 mm^3^ from 7 to 11 days in AB12 with the addition of αPD-1 (*P* = 0.028, Fig. 2C) and an increase from 13 to 17 days in AE17O (*P* < 0.001, Fig. 2F). Furthermore, treatment with αPD-1 decreased the level of PD-1 on AB12-infiltrating CD4 and CD8 T cells at 2 days after a single dose of αPD-1 (*P* < 0.001, Fig. 2G and H).

### TI amplifies immunosuppressive characteristics of the mesothelioma microenvironment

**Figure 3:**
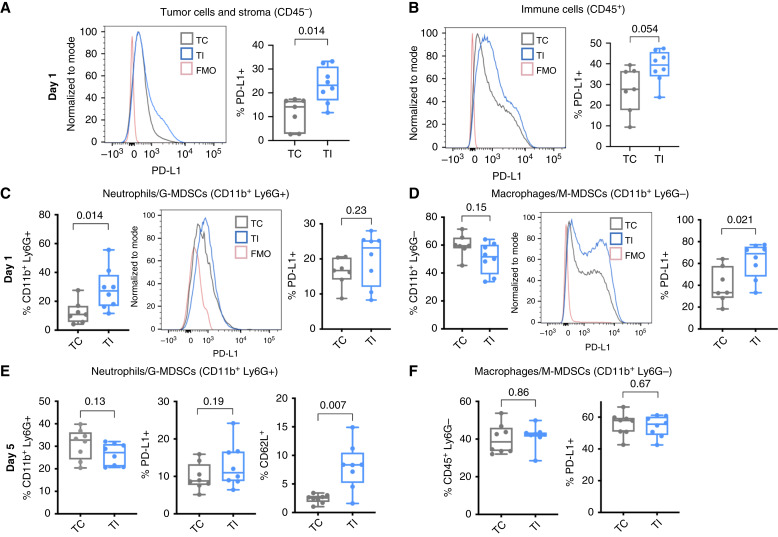
TI upregulates PD-L1 expression on AB12 intratumoral myeloid cells. **A,** In AB12 tumors, all CD45^−^ cells, a combination of tumor and stromal cells, were significantly increased in PD-L1 expression 24 hours after TI compared with TC (*P* = 0.014). **B,** For all CD45^+^ immune cells, PD-L1 levels slightly increased 24 hours after TI compared with TC (*P* = 0.054). **C,** The percentage of intratumoral CD11b^+^ Ly6G^+^ cells (as % of all CD45^+^ cells) was increased by TI (*P* = 0.014); however, changes in the levels of PD-L1 on these cells were not detected. **D,** The percentage of CD11b^+^ Ly6G^−^ myeloid cells was not significantly changed 24 hours after TI; however, TI tumors had significantly higher PD-L1 expression in this myeloid cell population (*P* = 0.021). For all 24-hour data, *n* = 7 tumors for TC and 8 tumors for TI. **E,** After 5 days, the numbers of CD11b^+^ Ly6G^+^ cells were about 30% of all CD45^+^ cells for both TC and TI tumors. At this time point, PD-L1 expression remained unchanged. However, CD62L expression (L-selectin) significantly increased for mice that received TI (*P* = 0.007). **F,** For CD11b^+^ Ly6G^−^ cells, no differences were observed in population numbers or PD-L1 levels. For all 5-day data, *n* = 8 for TC and *n* = 8 for TI. Comparisons were made using a Mann–Whitney test. FMO, fluorscence minus one.

To elucidate why αPD-1 improves local control of tumor after a surgical procedure, we investigated the effects of TI on the tumor microenvironment via flow cytometry (gating strategies shown in Supplementary Fig. S3). PD-1 (CD279) is a transmembrane receptor expressed mainly on activated T cells and B cells, as well as NK cells, and occasionally on myeloid cells (37, 38). Its ligands, PD-L1 and PD-L2, are expressed on a wider variety of cell types—leukocytes (particularly myeloid cells), nonhematopoietic cells, and nonlymphoid tissues, including tumors (39). Our data identify a broad trend toward an increase in PD-L1 expression on all immune cells (CD45^+^) in the AB12 tumor microenvironment and a significant increase (*P* = 0.014) in PD-L1–positive tumor and stromal cells (CD45^−^) at 1 day after TI versus TC (Fig. 3A and B). At this time point, neutrophils/G-MDSCs (CD11b^+^ Ly6G^+^) increase substantially (*P* = 0.014) in number with minimal changes in their PD-L1 expression (Fig. 3C), whereas PD-L1 expression is significantly increased (*P* = 0.021) in populations of primarily macrophages/monocytic MDSCs (M-MDSC; CD11b^+^ Ly6G^−^; Fig. 3D). At day 5 after TI, no change was detected in the percentage of tumor-associated Ly6G^+^ or Ly6G^−^ cells or their PD-L1 levels (Fig. 3E and F), although there is a slightly higher proportion of Ly6C^+^ M-MDSCs after TI compared with TC (22% in TI vs. 16% in TC; *P* = 0.041; Supplementary Fig. S4A). Interestingly, at day 5 after TI, neutrophils/G-MDSCs have a marked increase in CD62L (L-selectin) compared with those from TC (2% vs. 8%, respectively), representing a population recently mobilized to the tumor (*P* = 0.007, Fig. 3E; refs. 40, 41). These CD62L^+^ cells also have significantly higher PD-L1 expression (20% in TI vs. 14% in TC; *P* = 0.01; Supplementary Fig. S4B).

**Figure 4:**
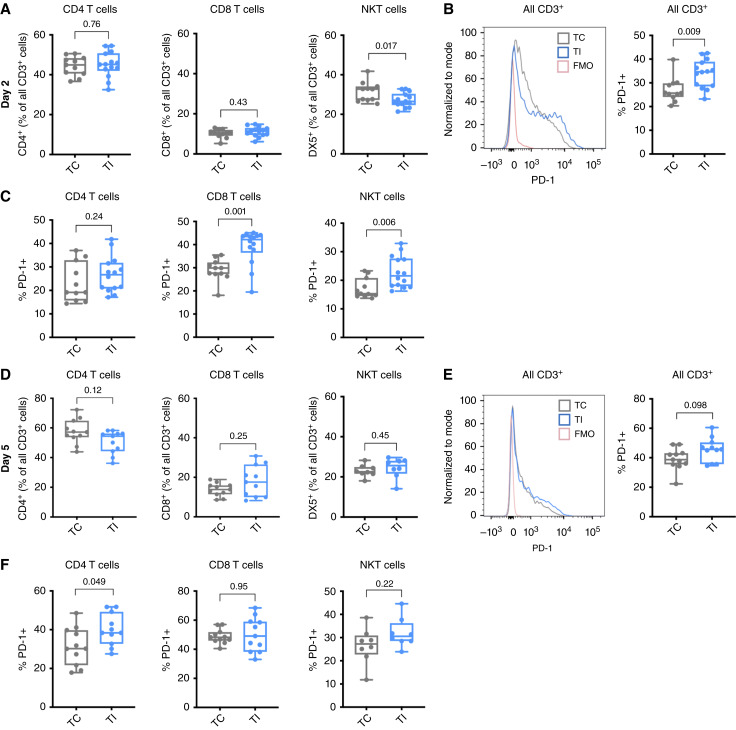
TI increases PD-1 expression on AB12 intratumoral T cells. **A,** In AB12 tumors, 2 days after TI, the fraction of CD4 and CD8 T cells (as a percentage of all CD3^+^ cells) was not significantly different between TI (*n* = 11) and TC (*n* = 14). However, the levels of DX5+ (NKT cells) decreased slightly but significantly in TI (*P* = 0.017). **B,** For all CD3^+^ cells, the levels of PD-1 were significantly upregulated by TI compared with TC (*P* = 0.009). **C,** For individual T-cell populations, PD-1 was significantly increased in CD8^+^ (*P* = 0.001) and DX5+ (*P* = 0.006) T cells after TI compared with TC. **D,** After 5 days, TC and TI tumors have similar numbers of CD8, CD4, and DX5+ T cells. For CD4 and CD8 T cells, *n* = 11 for TC and *n* = 11 for TI. For NKT cells, *n* = 8 for TI and *n* = 8 for TC. **E,** For all CD3^+^ cells, significant differences in median PD-1 levels were not detected at the 5-day time point (*P* = 0.098). **F,** CD4^+^ T cells had significantly higher PD-1 expression 5 days after TI (*P* = 0.049). Differences in PD-1 expression of CD8 and DX5 T cells at 5 days after TI were small and not statistically significant. Comparisons were made using a Mann–Whitney test. FMO, fluorscence minus one.

Next, T-cell populations and their PD-1 expression after TI were evaluated. At 2 days after TI in AB12 tumors, CD8/CD4 T-cell numbers did not significantly change relative to TC, although we find a small but significant decrease in NKT cells (*P* = 0.017, Fig. 4A). PD-1 expression significantly increased on all CD3^+^ cells in TI compared with TC (*P* = 0.009, Fig. 4B), with most changes occurring in CD8^+^ T cells (*P* = 0.001) and NKT cells (*P* = 0.006, Fig. 4C). By day 5, all T-cell types have similar proportions in TI compared with TC (Fig. 4D), and all CD3^+^ cells trend toward higher PD-1 expression after TI compared with TC, although these results were not statistically significant (*P* = 0.098, Fig. 4E). Nonetheless, CD4^+^ T cells gain significantly more PD-1 expression (*P* = 0.049, Fig. 4F) at this time point. Overall, in the TI model, we identified increases in neutrophils/G-MDSCs, as well as increases in their PD-L1 expression, suggesting a more immunosuppressive environment, occurring alongside the increased expression of PD-1 on T cells.

### PDT partially overcomes TI-induced immune checkpoint activity

**Figure 5:**
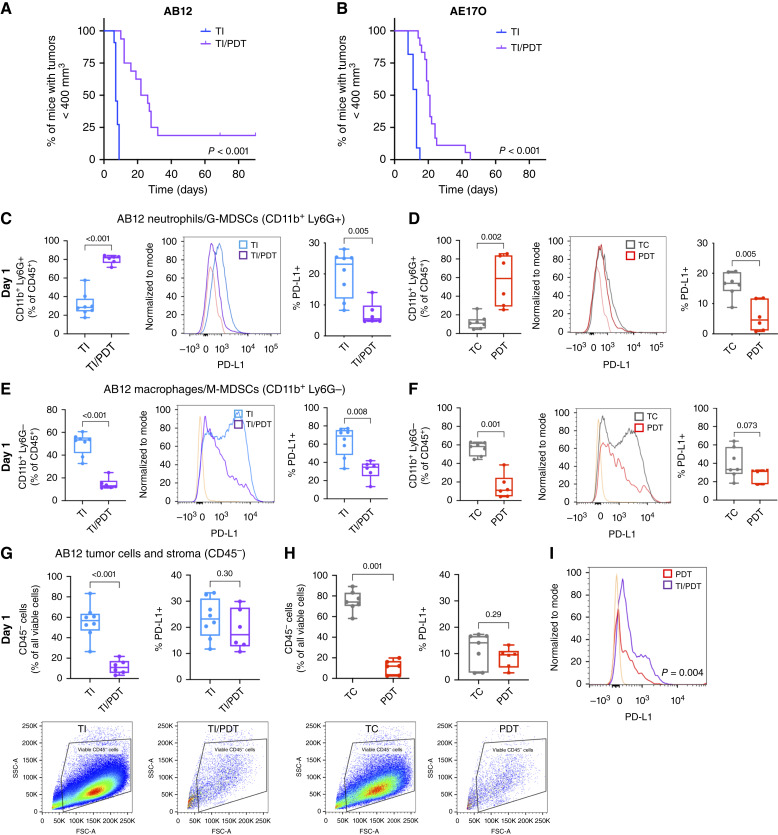
PDT decreases PD-L1 expression and significantly reduces tumor growth after TI. TI/PDT-treated mice had significantly longer tumor growth times (to 400 mm^3^) than TI mice for AB12 (*P* < 0.001, **A**) and AE17O (*P* < 0.001, **B**), with medians of 7 days for TI vs. 24 days for TI/PDT for AB12 and 13 days for TI vs. 20.5 days for TI/PDT for AE17O. In AB12 mice, *n* = 11 for TI and *n* = 16 for TI/PDT. In AE17O mice, *n* = 11 for TI and n = 18 for TI/PDT. **C,** CD11b^+^ Ly6G^+^ cells were significantly increased after TI/PDT (*n* = 6) compared with TI (*n* = 8) in AB12 tumors (*P* < 0.001), and their PD-L1 expression was significantly decreased following PDT (*P* = 0.005). **D,** Tumors treated with PDT (*n* = 6) similarly had increased Ly6G^+^ cells (*P* = 0.002) and decreased PD-L1 expression (*P* = 0.005) compared with TCs (*n* = 7). **E,** PDT after TI significantly decreased the proportion of Ly6G^−^ cells (*P* < 0.001) and led to a significant drop in their PD-L1 levels (*P* = 0.008). **F,** The proportion of CD11b^+^ Ly6G^−^ cells as a subset of CD45^+^ immune cells decreased significantly after PDT (*P* = 0.001), and their PD-L1 levels were lower on average but not significantly so (*P* = 0.073). **G,** For CD45^−^ cells, TI/PDT led to a significant drop in the percentage of viable CD45^−^ cells (*P* < 0.001) but did not alter PD-L1 expression (*P* = 0.30). **H,** PDT of CD45^−^ cells also significantly decreased viability (*P* = 0.001), but no significant differences in PD-L1 expression of these cells were observed (*P* = 0.29). Representative gates of viable CD45^−^ cells are shown below for each condition. **I,** Viable CD45^−^ cells after TI/PDT had significantly higher levels of PD-L1 than CD45^−^ cells after PDT alone (*P* = 0.004). Tumor responses (growth to 400 mm^3^) were compared using log-rank (Mantel–Cox) tests, and all other comparisons were made using a Mann–Whitney test. FSC-A, forward scatter area; SSC-A, side scatter area.

We previously found that TI alters PDT-driven antitumor immunity and tumor response when compared with PDT as a standalone alone therapy in AB12 and AE17O tumors (Supplementary Fig. S5A and S5B; ref. 20). One potential explanation for this is an increase in G-MDSCs after TI/PDT compared with PDT, evaluated by the ability of G-MDSCs to inhibit T-cell proliferation (PDT vs. TI/PDT *P* < 0.001 for CD4 and CD8 T cells; Supplementary Fig. S5C). Importantly, however, PDT of the inflammatory TI-altered tumor environment still does produce some effect, significantly slowing tumor growth in both AB12 (Fig. 5A; *P* < 0.001) and AE17O (Fig. 5B; *P* < 0.001) tumor models relative to TI alone. As our earlier studies pointed to the involvement of splenic G-MDSCs in limiting systemic antitumor immunity (20), we next evaluated PD-L1 activity in the myeloid subset of the AB12 tumor microenvironment after PDT.

As first responders in inflammation, neutrophils are critical to the development of antitumor immunity following PDT. Neutrophil infiltration in the tumor begins within an hour of PDT and is substantial at 24 hours, with more than 30% of all immune cells in the tumor microenvironment staining positive for Ly6G (42, 43). When applied after TI, Photofrin-PDT (135 J/cm^2^) increases the levels of KC/CXCL1, MIP2/CXCL2, IL-6, and JE/CCL2 and decreases IP-10/CXCL10, MIG/CXCL9, TIMP-1, and IL-1Ra compared with TI alone (Supplementary Fig. S6). Many of the increased cytokines are chemoattractants or activators for neutrophils, macrophages, and other myeloid cells and were previously shown to be upregulated by PDT (44). Indeed, in this AB12 model, TI/PDT promotes an accumulation of intratumoral neutrophils/G-MDSCs after 24 hours (*P* < 0.001; Fig. 5C). Similar to that previously reported for AE17O (23), neutrophils/G-MDSCs also increase after PDT alone compared with AB12 TCs (*P* = 0.002; Fig. 5D). In conjunction with increases in neutrophils/G-MDSCs, the levels of TAMs/M-MDSCs are proportionally decreased at this time point (TI/PDT vs. TI; *P* < 0.001 and PDT vs. TC; *P* = 0.001; Figs. 5E and F).

PDT also decreases the levels of PD-L1 in AB12 intratumoral myeloid cells. At 24 hours after PDT, PD-L1 expression in Ly6G^+^ cells is significantly lower after TI/PDT compared with TI [Fig. 5C (right panels); *P* = 0.005] and significantly lower in PDT compared with TC alone [Fig. 5D (right panels); *P* = 0.005]. Similar results are found for Ly6G^−^ cells, in which PDT following TI significantly lowers PD-L1 expression [TI vs. TI/PDT; *P* = 0.008; Fig. 5E (right panels)]. PDT slightly but not significantly reduces PD-L1 in Ly6G^−^ cells compared with TC alone [*P* = 0.073; Fig. 5F (right panels)]. Likewise, our previous study in AE17O tumors found a decrease in PD-L1 in these populations after PDT compared with TC (23). However, although the number of viable CD45^−^ cells in AB12 tumors is markedly diminished for PDT and TI/PDT groups 24 hours after PDT (gating strategy shown in Supplementary Fig. S7), the levels of PD-L1 on these cells remain unchanged (Fig. 5G and H).

Taken together, these results suggest that although PDT can decrease the levels of PD-L1 in myeloid cells and kill most tumor cells, remaining tumor cells maintain higher levels of PD-L1 expression after TI/PDT compared with PDT (*P* = 0.004, Fig. 5I). This may contribute to a less effective antitumor immune response after TI/PDT compared with PDT alone.

### PD-1 blockade mitigates immunosuppressive effects of TI that is performed prior to PDT

**Figure 6:**
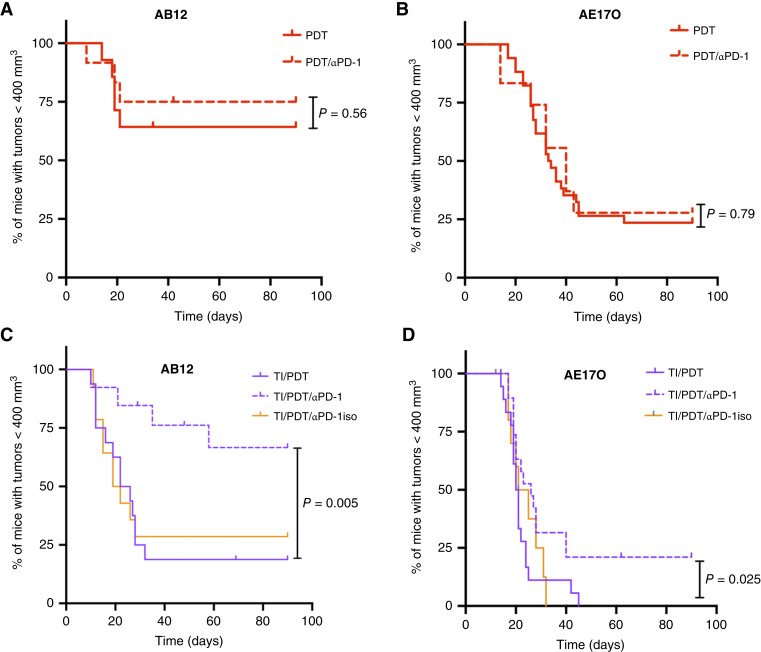
PD-1 blockade significantly decreases tumor growth rates in combination with TI/PDT. αPD-1 initiated 1 hour after PDT and continuing 2× weekly for 3 weeks did not significantly alter tumor growth rates. **A,** In AB12 mice, more than 50% of both PDT alone (*n* = 14) and PDT/aPD-1 (*n* = 12) had median survival (time to 400 mm^3^) greater than 90 days, and no differences between groups were detected (*P* = 0.56). **B,** In AE17O mice, median survivals of 33.5 days for PDT (*n* = 34) and 40 days for PDT/αPD-1 (*n* = 12) were similar (*P* = 0.79). **C,** In AB12 tumor–bearing mice that received TI prior to PDT, αPD-1 significantly decreased the rate of tumor regrowth (*P* = 0.005), with a median survival >90 days for TI/PDT/αPD-1 (*n* = 13) and 24-day median survival in TI/PDT (*n* = 16). TI/PDT/αPD-1 isotype control (*n* = 13) was also significantly different from TI/PDT/αPD-1 (*P* = 0.025) and similar to TI/PDT (*P* = 0.87). **D,** In AE17O tumor–bearing mice that received TI prior to PDT, αPD-1 also significantly decreased the rate of tumor regrowth (*P* = 0.025), with a median survival of 20.5 days for TI/PDT (*n* = 18) and 26 days for TI/PDT/αPD-1 (*n* = 20). In all tumor responses, vertical dashes indicate animals that were censored due to tumor ulceration or metastasis. Groups treated with an isotype for αPD-1 are plotted in pale orange. Tumor responses were compared using log-rank (Mantel–Cox) tests.

As an adjuvant therapy, ICB tends to be more effective when combined with a cytotoxic therapy that improves antitumor immune response, such as PDT. As presented above (see Fig. 2), PD-1 blockade slightly but significantly increased survival after TI alone in both AB12 and AE17O tumor models. Yet, in mice treated with PDT alone, αPD-1 provided no additional benefit to CR in both models (Fig. 6A and B). It seems likely in this scenario that PDT has already maximized tumor response by directly killing the tumor cells and attenuating PD-1/PD-L1 interactions with myeloid cells (see Fig. 5D and F). Although PDT does not significantly alter PD-L1 expression in viable tumor cells (see Fig. 5G), PD-L1 levels in CD45^−^ cells are already notably low in TC (see Fig. 5H vs. G) and thus simply remain low after PDT (without accompanying TI).

Still, in the clinic, PDT of PM is best delivered after tumor resection in order to minimize the bulk of disease prior to light delivery. In this context, we find that αPD-1 considerably improves survival after the combination of TI and PDT, returning overall CR to the level of PDT alone in both AB12 and AE17O tumors (Fig. 6C and D). Here, PD-1 blockade improves local control of tumor in combined modality treatments for PM that include surgery.

### Ly6G^+^ and CD8^+^ cells are required for efficacy of PD-1 blockade after TI/PDT

**Figure 7:**
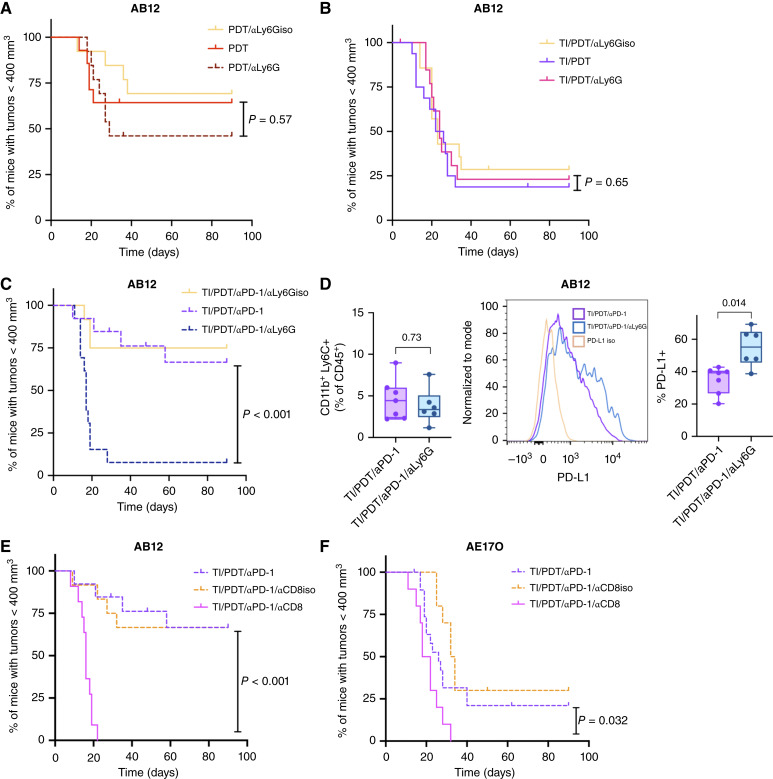
Effective PD-1 blockade requires Ly6G^+^ myeloid cells and CD8^+^ T cells. **A,** In AB12 tumors, αLy6G initiated 1.5 hours after PDT does not significantly change tumor response (*P* = 0.57; PDT/αLy6G shown as dark red dotted line). For PDT, *n* = 14 and for PDT/αLy6G, *n* = 13. PDT/αLy6G isotype control (*n* = 13) is also not significantly different from PDT/αLy6G (*P* = 0.17) or from PDT alone (*P* = 0.65). **B,** In TI/PDT-treated mice, Ly6G depletion did not significantly impact the time for tumors to regrow to 400 mm^3^ (TI/PDT/αLy6G, *n* = 14, shown as pink line) The TI/PDT/αLy6G isotype control group (*n* = 14) is similar (*P* = 0.81 compared with TI/PDT/αLy6G and *P* = 0.42 compared with TI/PDT). **C,** In TI/PDT-treated mice that also received αPD-1, Ly6G depletion led to a rapid and significant decrease in mice with tumors <400 mm^3^ (*P* < 0.0002; TI/PDT/αPD-1/αLy6G, *n* = 13, shown as dark blue dotted line). The TI/PDT/αPD-1/αLy6G isotype control group (*n* = 12) is similar to TI/PDT/αPD-1 (*P* = 0.80) and has significantly longer survival than TI/PDT/αPD-1/αLy6G (*P* < 0.001). **D,** Flow cytometric analysis of tumor-infiltrating lymphocytes 1 day after TI/PDT/αPD-1 demonstrated that Ly6G depletion after TI/PDT/αPD-1 did not significantly change the percentage of Ly6C+ cells in the tumor (*P* = 0.73, left, *n* = 7 tumors for TI/PDT/αPD-1 and *n* = 6 tumors for TI/PDT/αPD-1/αLy6G). However, Ly6G depletion led to a significant increase in the PD-L1 expression on the Ly6C^+^ cells (*P* = 0.014, right). **E,** In AB12 tumors, CD8 depletion (TI/PDT/αPD-1/αCD8, *n* = 11, shown as pink lines) led to rapid tumor regrowth (*P* <0 .001 compared with TI/PDT/αPD-1). The TI/PDT/αPD-1/αCD8 isotype control group (*n* = 12) is similar to TI/PDT/αPD-1 (*P* = 0.89) and has significantly longer survival than TI/PDT/αPD-1/αCD8 (*P* < 0.001). **F,** Similarly in AE17O tumors, CD8 depletion (TI/PDT/αPD-1/αCD8, *n* = 10) also led to rapid tumor regrowth (*P* = 0.033 compared with TI/PDT/αPD-1). The TI/PDT/αPD-1/αCD8 isotype control group (*n* = 10) is similar to TI/PDT/αPD-1 (*P* = 0.24) and has significantly longer survival than TI/PDT/αPD-1/αCD8 (*P* < 0.001). Groups treated with an isotype for αLy6G or αCD8 are plotted in pale orange. Tumor responses are compared using log-rank (Mantel–Cox) tests, and all other statistical tests are by Mann–Whitney.

Neutrophils are critical to immune responses to PDT (18), and we have identified dramatic changes in Ly6G^+^ cell populations with PDT treatment (see Fig. 5). To test whether these cells were required for PDT response, neutrophils were depleted with αLy6G in the AB12 model. In untreated control tumors, this resulted in an intratumoral drop in neutrophil levels from ∼40% of CD45^+^ cells to less than 20%, which was maintained at day 7 (Supplementary Fig. S8D and S8E). In conjunction with PDT alone, Ly6G depletion initiated 1.5 hours after PDT slightly worsened but did not significantly decrease PDT efficacy (*P* = 0.57; see Fig. 7A). After TI/PDT, our prior results suggested that splenic G-MDSCs could contribute to reductions in antitumor immunity (20), and tests of suppressive function of these cells reveal that they inhibit T-cell proliferation (Supplementary Fig. S5C). However, Ly6G depletion in conjunction with TI/PDT has no effect on therapeutic efficacy (Fig. 7B). From this, we expect that the intratumoral Ly6G^+^ population in this set of treatment conditions is likely a mix of protumor and antitumor phenotypes, making their collective depletion irrelevant to tumor response.

In the context of ICB, depletion of neutrophils/G-MDSCs is published to improve outcomes (45–47). Conversely, in study of the contribution of neutrophils/G-MDSCs to treatment with TI/PDT and PD-1 blockade, we find drastic reductions in treatment efficacy after depleting Ly6G^+^ cells (TI/PDT/αPD-1 vs. TI/PDT/PD-1/αLy6G; *P* < 0.001, Fig. 7C) in AB12 mice. Ly6G depletion did not significantly change the percentage of Ly6C^+^ cells (*P* = 0.73, Fig. 7D) at 24 hours after TI/PDT/αPD-1 treatment. However, Ly6G depletion did significantly increase PD-L1 expression on these cells [*P* = 0.014, Fig. 7D (right panels)]. Similar increases in PD-L1 expression in Ly6C^+^ cells were detected after Ly6G depletion for TI/PDT conditions (*P* = 0.005, Supplementary Fig. S9). Therefore, after Ly6G depletion, the proportion of myeloid cells with a suppressive phenotype was increased. These data show neutrophils to be required in αPD-1–treated mice for the establishment of antitumor immunity following surgery and PDT, and they support a role for TI-induced myeloid expression of PD-L1 in driving tumor response to this treatment.

Lastly, we examined the overarching contribution of antitumor immunity to the favorable treatment outcome produced by PD-1 blockade added to TI/PDT. Toward this goal, we evaluated the effects of CD8^+^ T-cell depletion on tumor response to TI/PDT/αPD-1. Antitumor immunity driven by PDT is mediated by CD8^+^ T cells, and CD8 depletion was previously shown to diminish the long-term effects of PDT (20). In the present study, the addition of αCD8 to TI/PDT/αPD-1 leads to rapid tumor regrowth and wholly removes the beneficial effect of PDT and of PD-1 inhibition after TI for both AB12 tumors (TI/PDT/αPD-1 vs. TI/PDT/αPD-1/αCD8; *P* < 0.001; Fig. 7E) and AE17O tumors (TI/PDT/αPD-1 vs. TI/PDT/αPD-1/αCD8; *P* = 0.032; Fig. 7F). We conclude from these findings that the generation of antitumor immunity is essential to the control of tumors provided by combinations of PD-1 blockade with TI/PDT.

## Discussion

Surgery is regularly performed for PM in combination with other treatments, including radiotherapies. Circulating and intratumoral cytokines increase rapidly during surgery with elevated levels of IL-6 and IL-1Ra in plasma, pleural fluid, and tissue studied as markers of surgical stress after thoracic surgery in patients with cancer (48–50). Our trial using surgical debulking and intraoperative PDT also demonstrated significant increases in IL-6 signaling in PM tumor tissue and plasma and significant increases in IL-1 signaling and IL-1Ra in tumor tissue over the course of surgical debulking (Fig. 1; ref. 19). IL-6 is a pleiotropic cytokine that plays many roles in the tumor microenvironment: inducing immune checkpoint activation via PD-1/PD-L1 expression (51, 52), negatively regulating NK and effector T cells (12), and promoting functionally suppressive immune populations, such as MDSCs, which contribute to tumor progression and therapy resistance (53, 54). IL-1 signaling is also correlated with increased levels of PD-1 and expansion of MDSCs, and high IL-1β expression is associated with unfavorable outcomes in lung cancer (55, 56). Although we did not observe fluctuations in IL-1α/IL-1β transcription at the time points studied, Il-1β itself would be expected to peak earlier than transcriptional or cytokine fluctuations detected in our studies (49). IL-6 also promotes PD-L1 increases on macrophages and monocytes (57), which is predictive of inferior survival for patients with lymphoma (58). Overall, this dataset provides a valuable and novel resource of transcriptomic changes in tissue that occur during surgical insult, and observed increases in inflammation-related pathways are likely generalizable to other tumor types. Subsequent analyses of this dataset in our PM clinical trial patients will include the correlation of cytokine and transcriptomic biomarkers with therapeutic response and outcomes.

In murine studies, we found the surgical inflammation of TI to increase immune checkpoint expression in myeloid cells (as PD-L1) and in CD8, CD4, and NKT cells (as PD-1; Figs. 3 and 4). In the context of TI, PDT improves survival, with some mice achieving CR (Fig. 5A and B), but fails to completely overcome the surgical-induced immunosuppressive environment (19, 20). We found that although PDT depletes PD-L1 on intratumoral myeloid cells, it does not significantly alter PD-L1 levels on tumor cells. Nonetheless, tumor cells treated with PDT alone (no TI) have lower PD-L1 levels than those treated with TI/PDT (Fig. 5G–I). This occurs because TI increases PD-L1 levels in tumors prior to the introduction of PDT, and the surviving tumor cells maintain pre-PDT levels of PD-L1 expression (Fig. 3A).

In conjunction with the above findings, PD-1 blockade within the perioperative period (Fig. 6) dramatically potentiates antitumor immunity and significantly augments PDT efficacy. Although more work needs to be performed to evaluate predictive biomarkers of response to ICB, αPD-1 may be particularly effective here because ICB has shown most promise in tumors with high expression of immune checkpoints or tumors in which adjuvant therapies have increased immune cell infiltrate (59). Although PD-1 blockade is very effective after the combination of TI and PDT, it has minimal impact on other treatment conditions. For example, αPD-1 only slightly improves survival after TI. This is in alignment with the low response rate of many PM tumors to ICB (7), and recent meta-analyses of clinical trials for lung cancer also show greater survival benefit for ICB initiated in the neoadjuvant setting compared with adjuvant ICB alone (60).

Investigations in two mouse models found regimens incorporating PDT and αPD-1 to be slightly more effective in the AB12 compared with AE17O tumors, consistent with our earlier findings in the immunogenicity of these models (20). These differences may be explained by different genetic backgrounds and characteristics of the Balb/c (AB12 tumors) and C57/BL6 (AE17O tumors) mouse strains, including strain-dependent Th2 versus Th1 lymphocyte response (61) or by the phenotypes and behavior of the corresponding mesothelioma cell lines (33, 62). Irrespective of mouse strain, tumor response was significantly impeded with the addition of TI to PDT (20), and we have importantly now identified that PD-1 blockade maximizes PDT efficacy in both cases.

The presence of neutrophils in the tumor-draining lymph node and tumor has been shown to enhance antitumor immunity following PDT (17) but only at specific time points. The depletion of granulocytes/G-MDSCs immediately after PDT significantly limited therapy response, whereas delaying the depletion of these cells to 1 hour after PDT improves response (63), potentially due to a shift in tumor-resident neutrophils from an antitumor to an immunosuppressive function with time after PDT. Through Ly6G depletion at 1.5 hours after TI/PDT combined with adjuvant αPD-1 (Fig. 7), we find that in this setting (i.e., TI/PDT/αPD-1), Ly6G^+^ cells strongly promote antitumor activity. In the context of PD-1 blockade, the depletion of neutrophils could be compensated by the activation of other immune checkpoints or IL-10, leading to the exposure of more M-MDSC–suppressive activity (64). Indeed, we found that the depletion of Ly6G^+^ cells increased PD-L1 levels on M-MDSCs in support of an enhanced immunosuppressive role for these cells (Fig. 7; Supplementary Fig. S9). Although we did not observe major changes in the population levels of M-MDSCs after TI, we plan to examine M-MDSCs and TAMs in future studies of multimodal treatments for mesothelioma, both in mice and patients with PM from our clinical trials. The interaction of macrophages with PM tends to make them more suppressive, potentially leading to downregulation of T-cell response to the tumor (65). This suggests that TAMs may be useful cell types to target in the treatment of PM tumors.

The TI model represents both a strength and a limitation of our study. As a strength, it allowed for isolation of the effects of inflammation on treatment outcome without confounding response by changing the tumor volume. Yet, TI is unlikely to produce as large of an inflammatory insult as would result from the surgical excision of tissue. Moreover, we could not utilize orthotopic models of mesothelioma, which propagate tumors inside the intact ribcage, because of the need to incise tumors during survival surgeries. Nonetheless, we previously reported an increase in IL-6 after incising flank tumors (TI) in mice (19, 20) comparable with that of patients with PM after surgical debulking; in this study, we show that TI increases the levels of intratumoral IL-1Ra at a similar time point as surgical resection in our PM clinical trial (Fig. 1). Thus, despite these limitations, the data clearly show that TI promotes an immunosuppressive state and establishes the efficacy of adding αPD-1 to TI/PDT for overcoming the TI effect on PDT response. Similarly, Guisier and colleagues (66) described tumor debulking of murine lung cancer to improve anti–PD-1 efficacy.

Overall, these studies suggest that surgically mediated modulation of immune cell trafficking and functionality prior to PDT leads to a systemic immunosuppressive state with increased PD-L1/PD-1 immune checkpoint activation. Although PDT can overcome some aspects of this immunosuppressive state, surgery still limits PDT-induced antitumor immune response. Targeted inhibition of surgery-induced signaling via PD-1 blockade can counteract surgery’s immunosuppressive outcomes to promote PDT efficacy in the intraoperative setting. These findings are especially significant for surgical studies going forward, given the negative results of surgical resection without intraoperative adjuvant or immunomodulation in the recently reported MARS2 trial (67). Indeed, our clinical data suggest that surgically mediated inflammation may lead to increases in immune checkpoint signaling and immunosuppression that will need to be addressed in the design of multimodal therapies for PM.

## Supplementary Material

Supplemental figure legends Supplemental figure legends

Supplementary Figure 1 RNA sequencing experimental design

Supplementary Figure 2 Supplemental RNA sequencing analysis

Supplementary Figure 3 General flow cytometry gating strategies.

Supplementary Figure 4 TI increases the proportion of AB12 tumor MDSCs and PD-L1 levels

Supplementary Figure 5 TI performed prior to PDT limits PDT efficacy.

Supplementary Figure 6 PDT increases cytokines involved in innate immune cell migration and activation.

Supplementary Figure 7 Flow cytometry gating strategy for Figure 5 G and H

Supplementary Figure 8 PD1 and Ly6G depletion

Supplementary Figure 9 Ly6G after TI/PDT depletion increases PD-L1 expression on Ly6C+ MDSCs

Supplementary Table S1 Genes found by DESeq2 significantly associated with time of sample collection
