# Supplementary material for: PD-1 Blockade Mitigates Surgery-Induced Immunosuppression and Increases the Efficacy of Photodynamic Therapy for Pleural Mesothelioma
Source: Cancer Res Commun. 2025 May 23;5(5):841–56. doi: 10.1158/2767-9764.CRC-24-0571 (PMC12099492; doi:10.1158/2767-9764.CRC-24-0571)
Supplement: Supplemental figure legends [file crc-24-0571_supplemental_figure_legends_suppsfl.docx]

**SUPPLEMENTAL FIGURE LEGENDS**

**Supplemental Figure 1. RNA sequencing experimental design. (A)** RNA sequencing workflow. 1, 2: Multiple PM specimens were collected from each patient over the course of surgery (at timepoints according to the surgeon’s discretion), with time of removal recorded, and immediately flash-frozen in liquid nitrogen. All tissues from all patients were stored at -80°C until further processing. 3: RNA was extracted from frozen sections of PM specimens. 4: cDNA was generated, libraries were prepared, and sequencing was performed on an Illumina NexSeq500. 5: Computational analysis was performed using the DESeq2 algorithm with design testing the effect of time of specimen collection over the course of surgery without additional patient information. 6: DESeq2 normalized counts of differentially expressed (DE) genes were used for heatmap expression visualization. Figure made in Adobe Illustrator with lungs diagram modified from NIAID NIH BIOART Source (bioart.niaid.nih.gov/bioart/231). (**B**) Swimmer plot showing length of surgery (in minutes, grey bar) for each patient with timepoints of specimens collected that were used for RNA sequencing analysis.

**Supplemental Figure 2. (A)** Correlation of pathways’ changes between timepoints within patient versus overall sample correlation reveals that reported pathways are changed in a general manner and not affected significantly by patient-specific expression. On the x axis, each dot shows correlation of pathway expression differences in 52 pairs of samples taken from the same patients versus time difference between samples. On the y axis, each dot shows direct correlation with time with no patient pairing involved. This analysis revealed almost perfect concordance (correlation r = 0.937) between results of timepoints within patient versus overall sample correlation. **(B)** Abundance and distribution of immune cell infiltration estimated using CIBERSORT. The heatmap shows relative increase (purple) or decrease (orange) of estimated proportion of immune cells as surgery progresses. Specimens are sorted by time from initiation of surgery (green bar at top). Specimens from individual patients (1 per row, color coded) are marked at time of collection. No significant trends in immune cell populations were detected over the course of surgery.

**Supplemental Figure 3. General flow cytometry gating strategies. (A)** Example gating strategy for examination of T cells in an untreated AB12 tumor. **(B)** Example gating strategy for examination of myeloid cells in an untreated AB12 tumor.

**Supplemental Figure 4. TI increases the proportion of AB12 tumor MDSCs and PD-L1 levels (A)** At day 5 post-TI, there is a slightly higher proportion of CD11b+ Ly6C+ M-MDSCs (p = 0.041 by Mann-Whitney test) after TI compared to TC. The proportion of Ly6C+ cells that are PD-L1+ or CD62L+ is not significantly changed by TI. **(B)** At 5 days post-TI, Ly6G+ cells that are CD62L+ also have significantly higher median PD-L1 expression compared to TC (p = 0.01 by Mann-Whitney test).

**Supplemental Figure 5. TI performed prior to PDT limits PDT efficacy. (A)** In subcutaneous AB12 mouse mesothelioma tumors, PDT (red, n = 14) leads to significantly delayed tumor regrowth and higher survival rates compared to TI/PDT (purple, n = 16, p = 0.029). Median survival is 24 days for TI/PDT and exceeds 90 days for PDT alone. **(B)** Similarly, in subcutaneous AE17O mouse mesothelioma tumors, PDT (red, n = 34) leads to significantly delayed tumor regrowth and higher survival rates compared to TI/PDT (purple, n = 18, p = 0.029). Median survival 20.5 days for TI/PDT and 33.5 days for PDT alone. AE17O Kaplan-Meier plots comparing PDT and TI/PDT represent a comprehensive set of data building off our previous results (20). Tumor responses are compared using log-rank (Mantel-Cox) tests. **(C)** Ly6G+ G-MDSCs isolated from spleens 2 days after each treatment in AB12 tumor-bearing mice were incubated with CTV-stained naïve T cells at 1:4 and 1:8 ratios. Ly6G+ cells from mice receiving PDT significantly increased T cell proliferation compared to those from TC (p <0.001 for CD4 and CD8 T cells). Ly6G+ cells from mice receiving TI/PDT significantly decreased T cell proliferation compared to PDT (p < 0.001 for CD4 and CD8 T cells) and compared to TC (p = 0.01 for CD4 and p = 0.004 for CD8 T cells). Representative flow cytometry histograms for T cell CTV staining in each experimental condition are shown below.

**Supplemental Figure 6. PDT increases cytokines involved in innate immune cell migration and activation.** Mouse cytokine arrays were performed for AB12 tumors 24h after TI or TI/PDT. 5 tumors were combined for each condition. Quantification of spot intensity (averaged for 2 spots/cytokine) is shown below, with TI in blue and TI/PDT in purple. Plots show increased levels of KC/CXCL1, MIP2/CXCL2, IL-6, and JE/CCL2, and decreased levels IP-10/CXCL10, MIG/CXCL9, TIMP-1, and IL-1Ra after TI/PDT compared with PDT.

**Supplemental Figure 7. Flow cytometry gating strategy for Figure 5 G and H.** Gating for viable CD45- AB12 tumor/stromal cells remaining 1 day after each treatment condition.

**Supplemental Figure 8. PD1 and Ly6G depletion.** In AB12 tumor controls, αPD1 was injected at 2.5 mg/kg αPD1 (clone RMP1-14) followed by a second dose after 4 days. Dot plots **(A)** and histograms **(B)** for PD-1 expression are quantified in **(C)**, with αPD1 leading to significantly less detectable PD-1 expression after 5 days (p = 0.029 by Mann-Whitney test). For αPD1, n = 4 and for αPD1 isotype control, n = 4. **(D)** In AB12 tumor controls, neutrophils were depleted with 350 ug αLy6G (clone 1A8), followed by a second dose after 4 days. **(E)** Flow cytometric analysis shows a significant decrease in the percentage of CD11b+ Ly6Cmid cells (p = 0.036 by Mann-Whitney test). For αLy6G, n = 5 and for αLy6G isotype control, n = 3.

**Supplemental Figure 9. Ly6G after TI/PDT depletion increases PD-L1 expression on Ly6C+ MDSCs.** In AB12 tumors, Ly6G depletion in conjunction with TI/PDT does not significantly change the percentage of Ly6C+ cells in the tumor at one day after treatment (p = 0.63, left panel). However, Ly6G depletion leads to a significant increase in PD-L1 expression on Ly6C+ cells after TI/PDT (p = 0.005, right panels). For TI/PDT, n = 5 tumors and for TI/PDT/αLy6G, n = 7 tumors. Statistical analyses by Mann-Whitney test.
