# Supplementary figures and images for: PD-1 Blockade Mitigates Surgery-Induced Immunosuppression and Increases the Efficacy of Photodynamic Therapy for Pleural Mesothelioma
Source: Cancer Res Commun. 2025 May 23;5(5):841–56. doi: 10.1158/2767-9764.CRC-24-0571 (PMC12099492; doi:10.1158/2767-9764.CRC-24-0571)

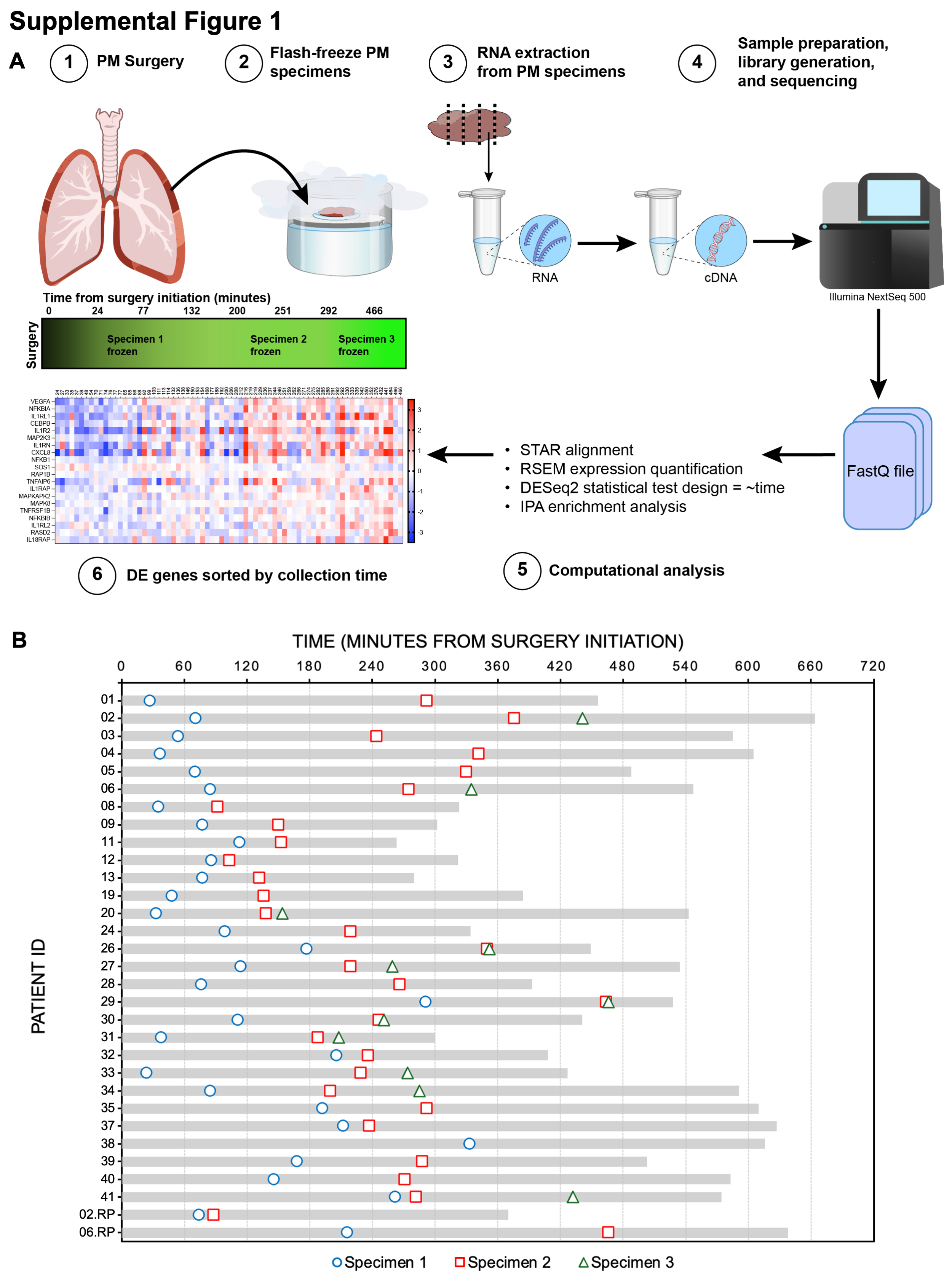

Supplement: Supplementary Figure 1 — RNA sequencing experimental design [file crc-24-0571_supplementary_figure_1_suppsf1.png]

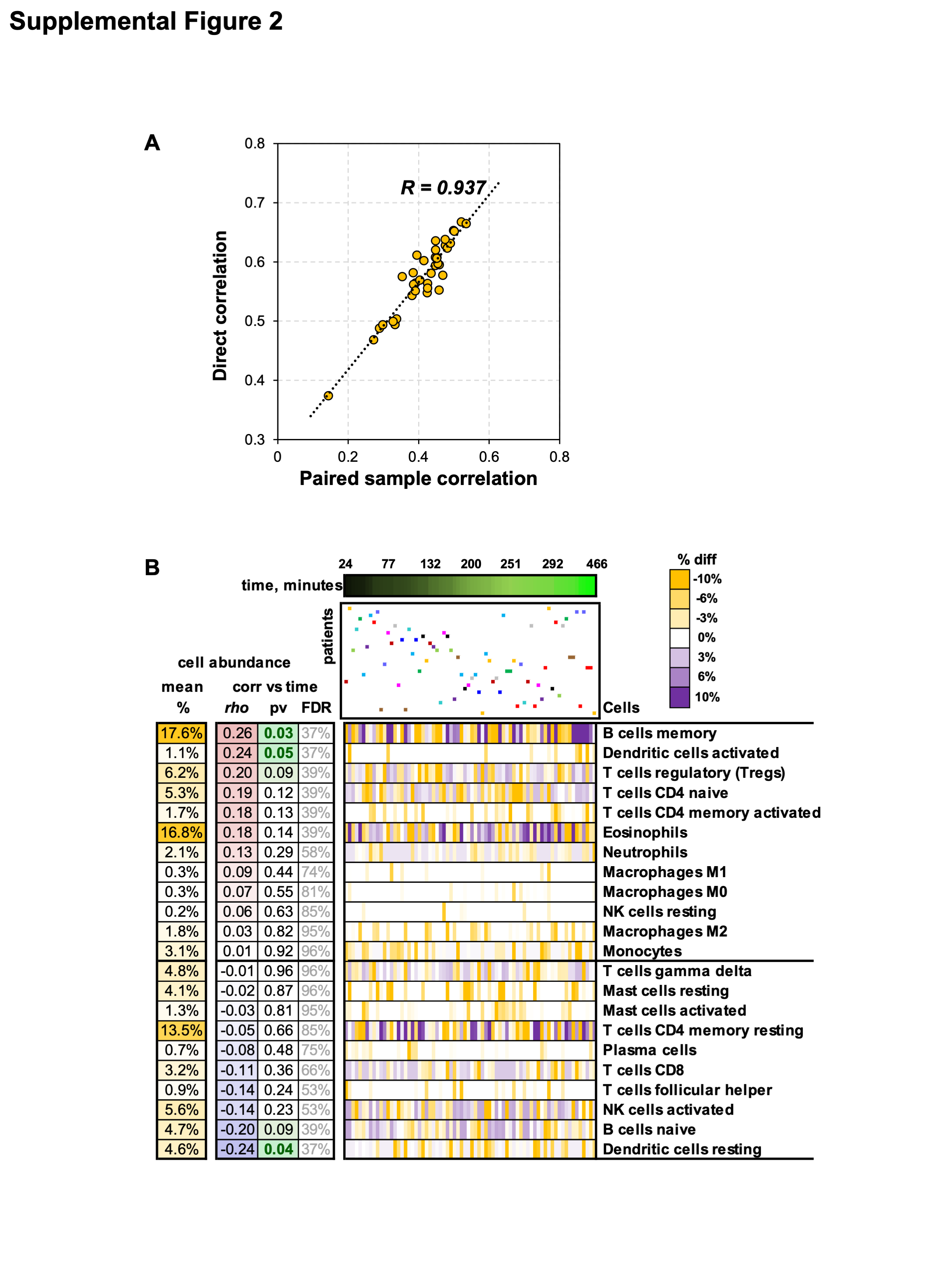

Supplement: Supplementary Figure 2 — Supplemental RNA sequencing analysis [file crc-24-0571_supplementary_figure_2_suppsf2.png]

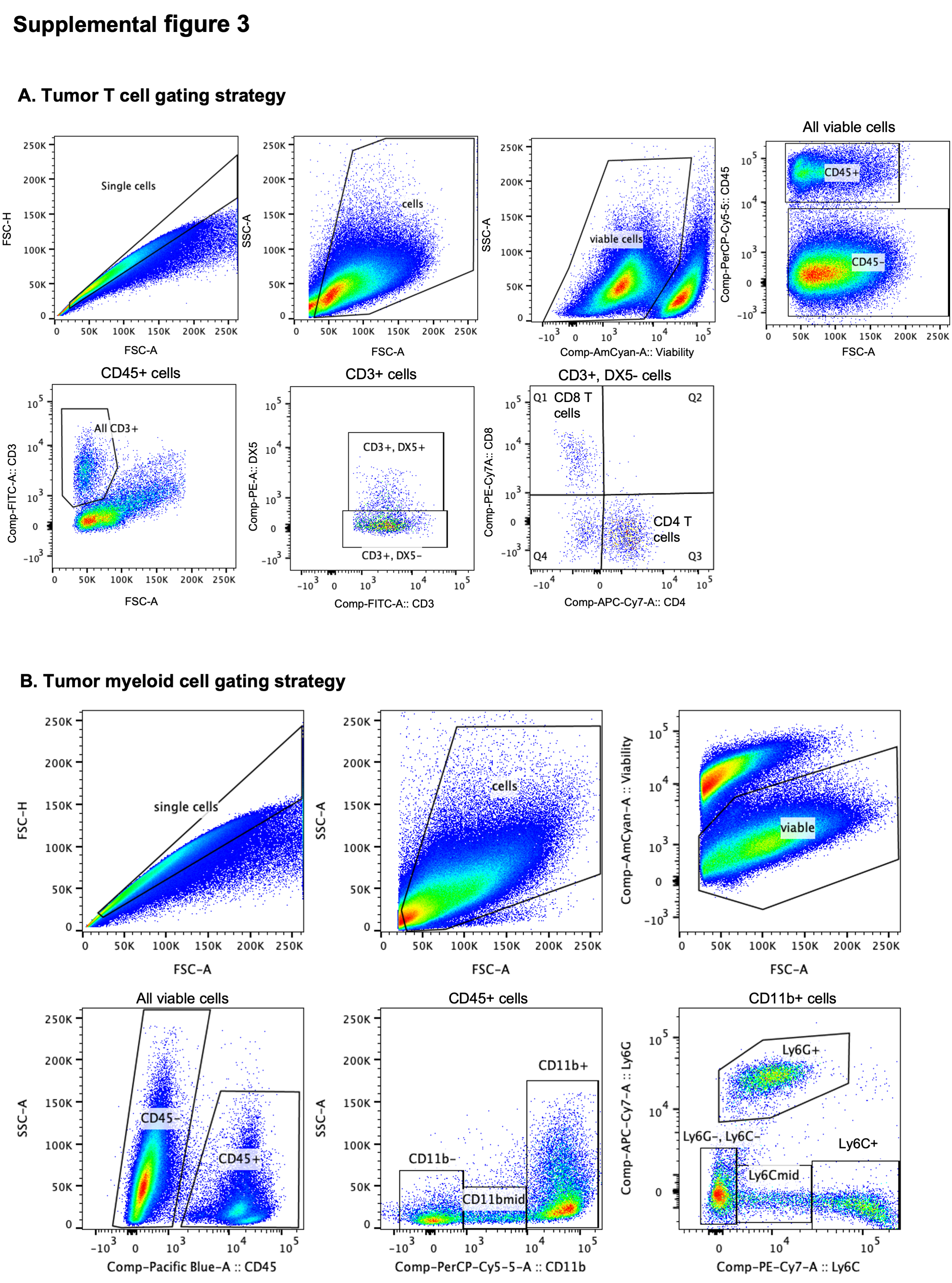

Supplement: Supplementary Figure 3 — General flow cytometry gating strategies. [file crc-24-0571_supplementary_figure_3_suppsf3.png]

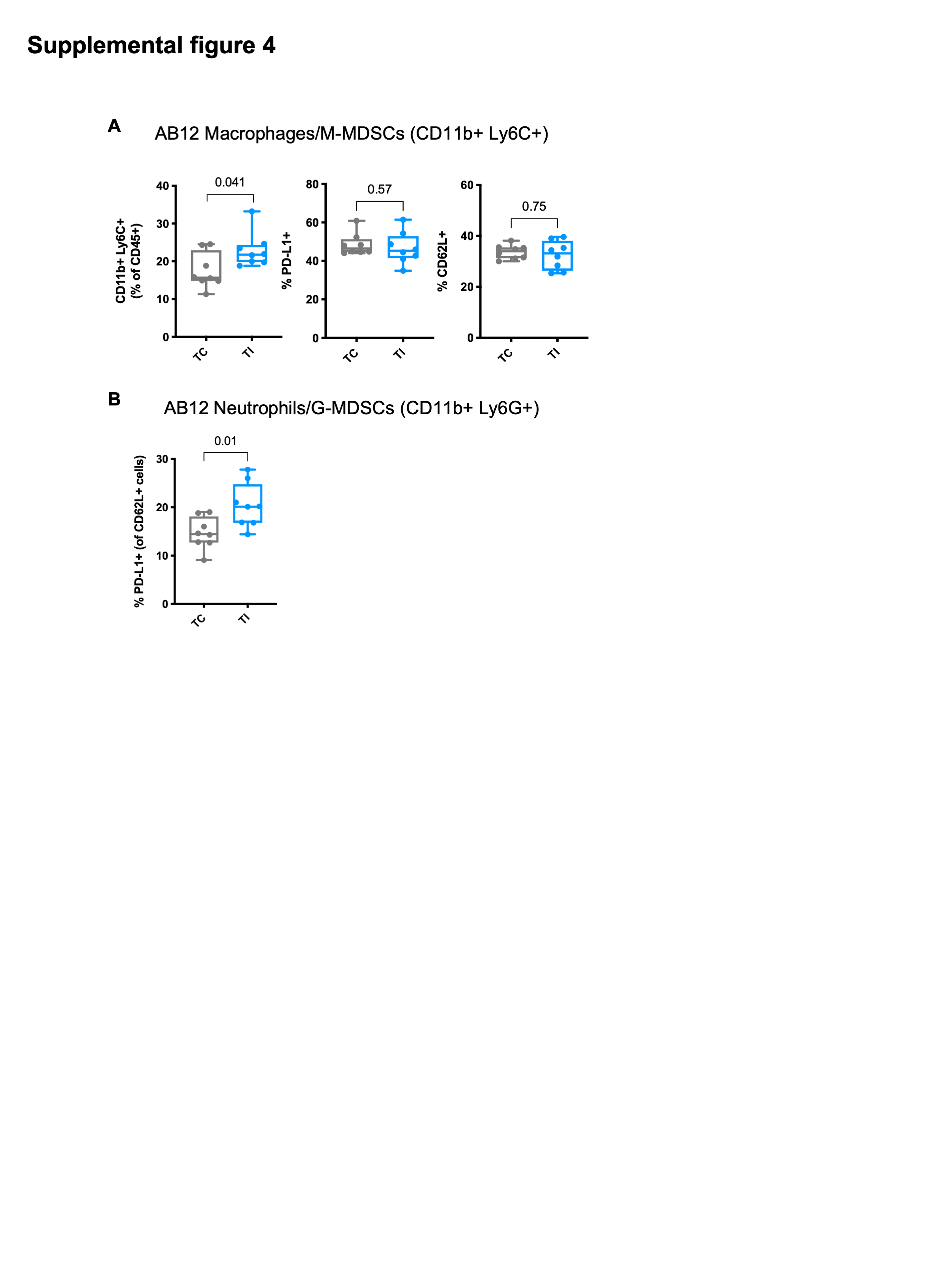

Supplement: Supplementary Figure 4 — TI increases the proportion of AB12 tumor MDSCs and PD-L1 levels [file crc-24-0571_supplementary_figure_4_suppsf4.png]

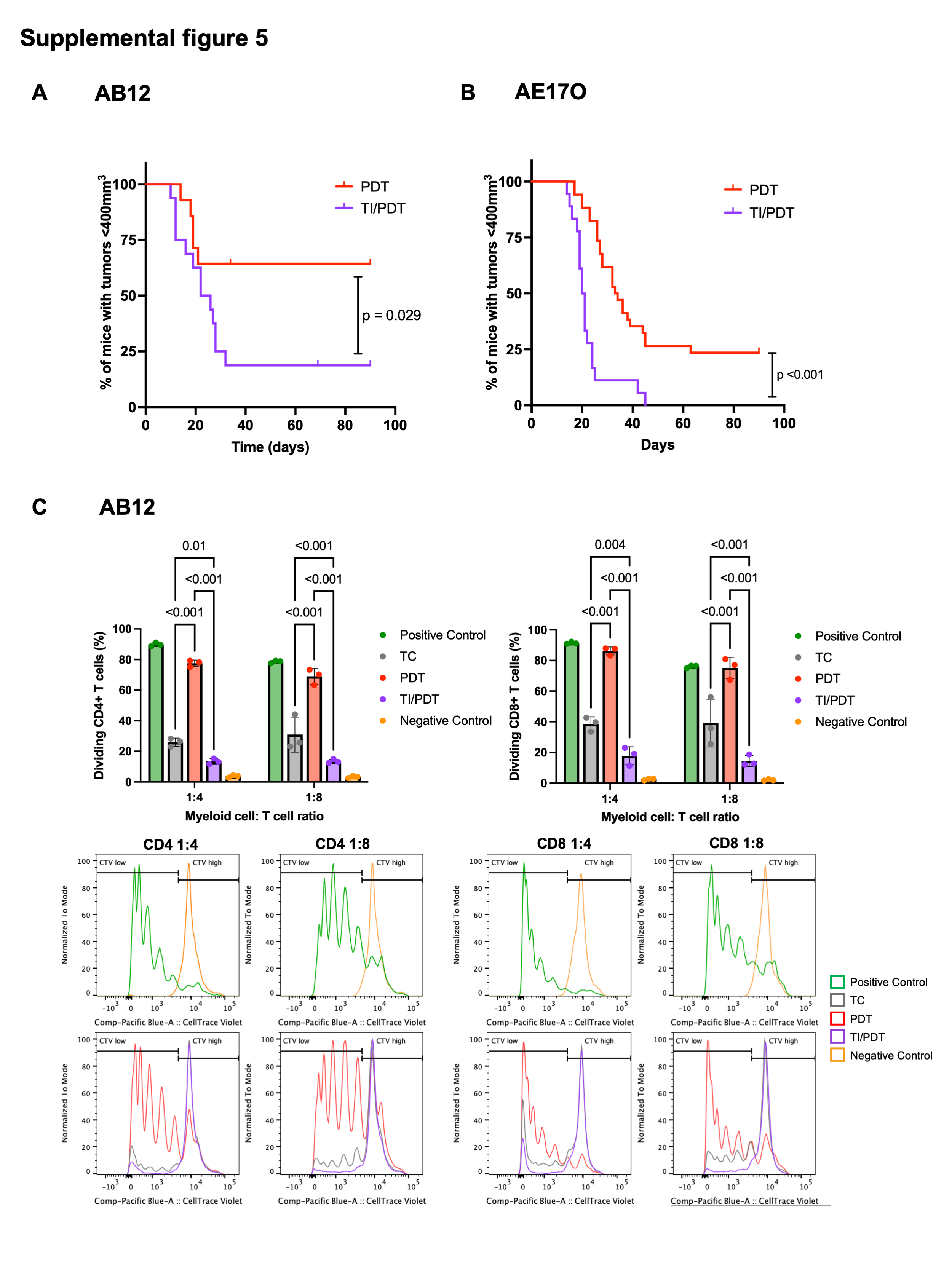

Supplement: Supplementary Figure 5 — TI performed prior to PDT limits PDT efficacy. [file crc-24-0571_supplementary_figure_5_suppsf5.png]

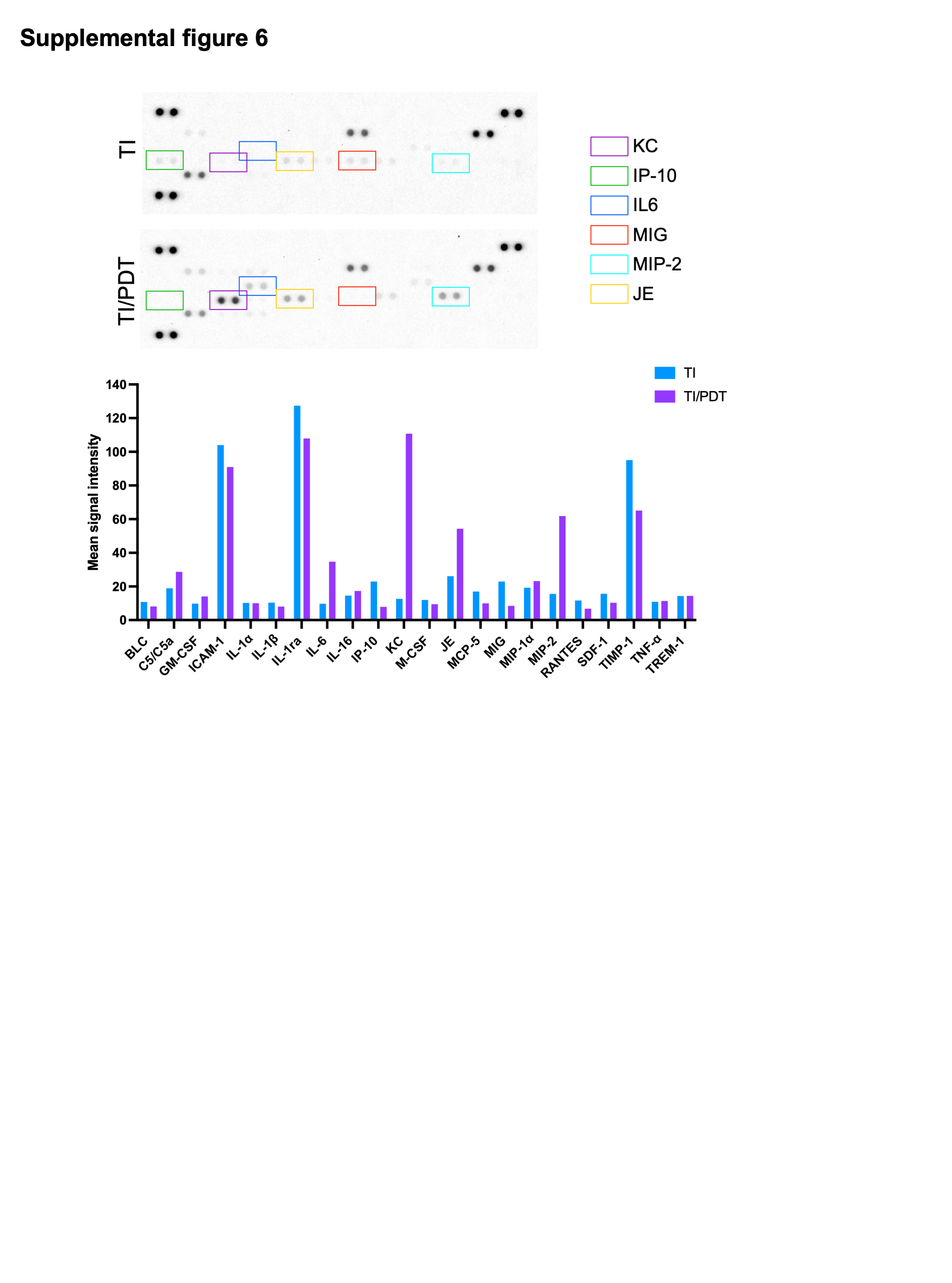

Supplement: Supplementary Figure 6 — PDT increases cytokines involved in innate immune cell migration and activation. [file crc-24-0571_supplementary_figure_6_suppsf6.png]

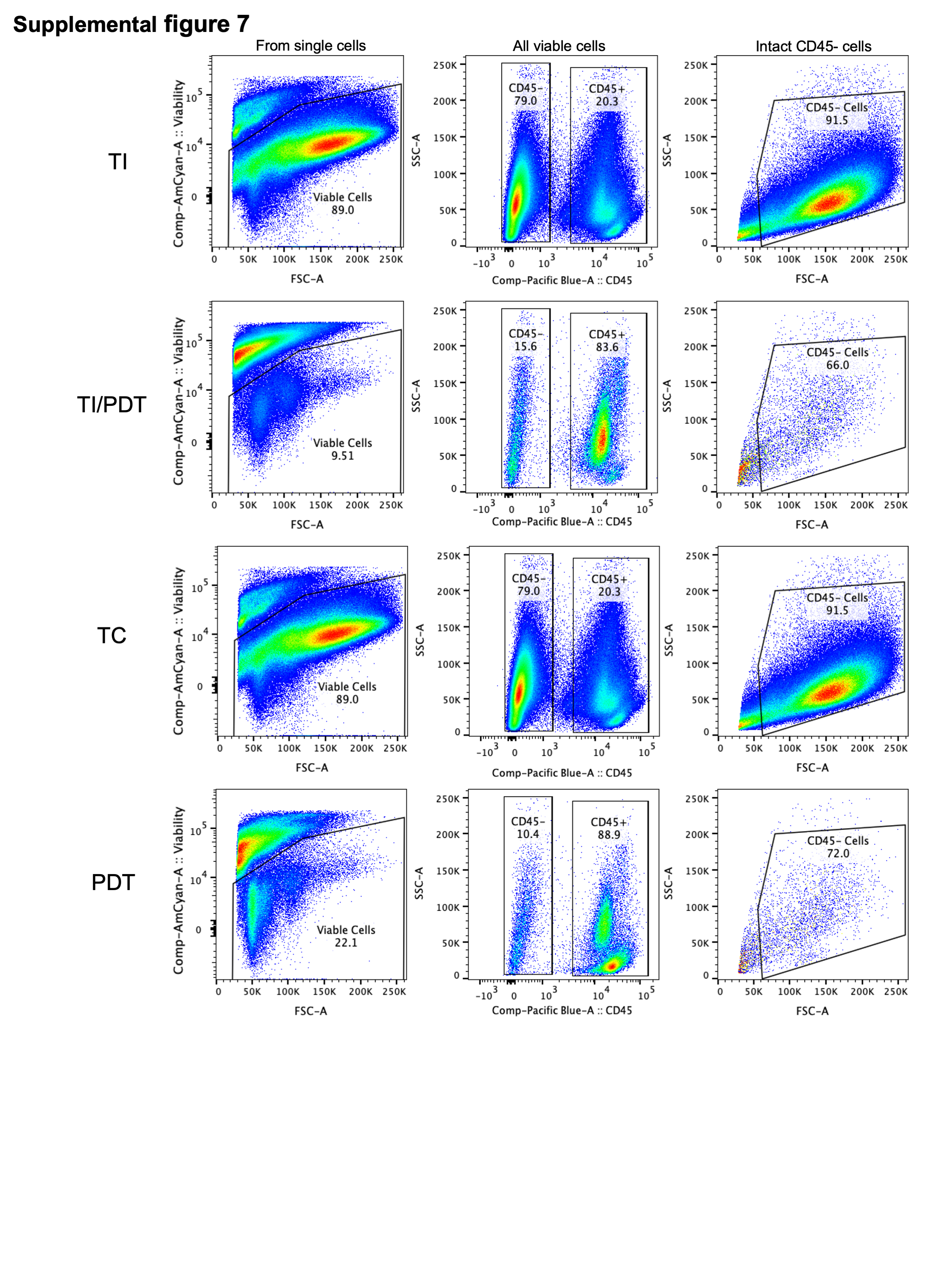

Supplement: Supplementary Figure 7 — Flow cytometry gating strategy for Figure 5 G and H [file crc-24-0571_supplementary_figure_7_suppsf7.png]

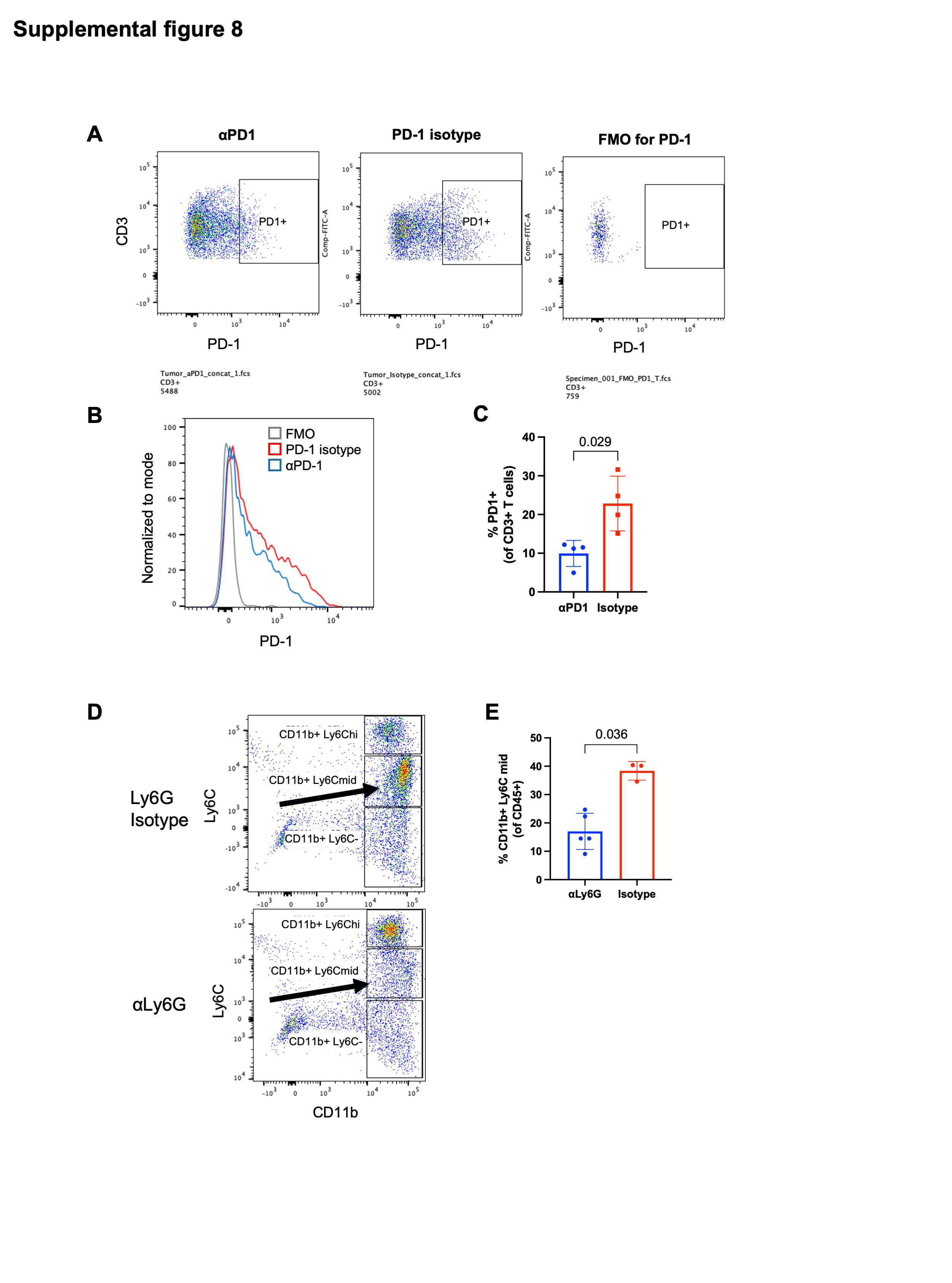

Supplement: Supplementary Figure 8 — PD1 and Ly6G depletion [file crc-24-0571_supplementary_figure_8_suppsf8.png]

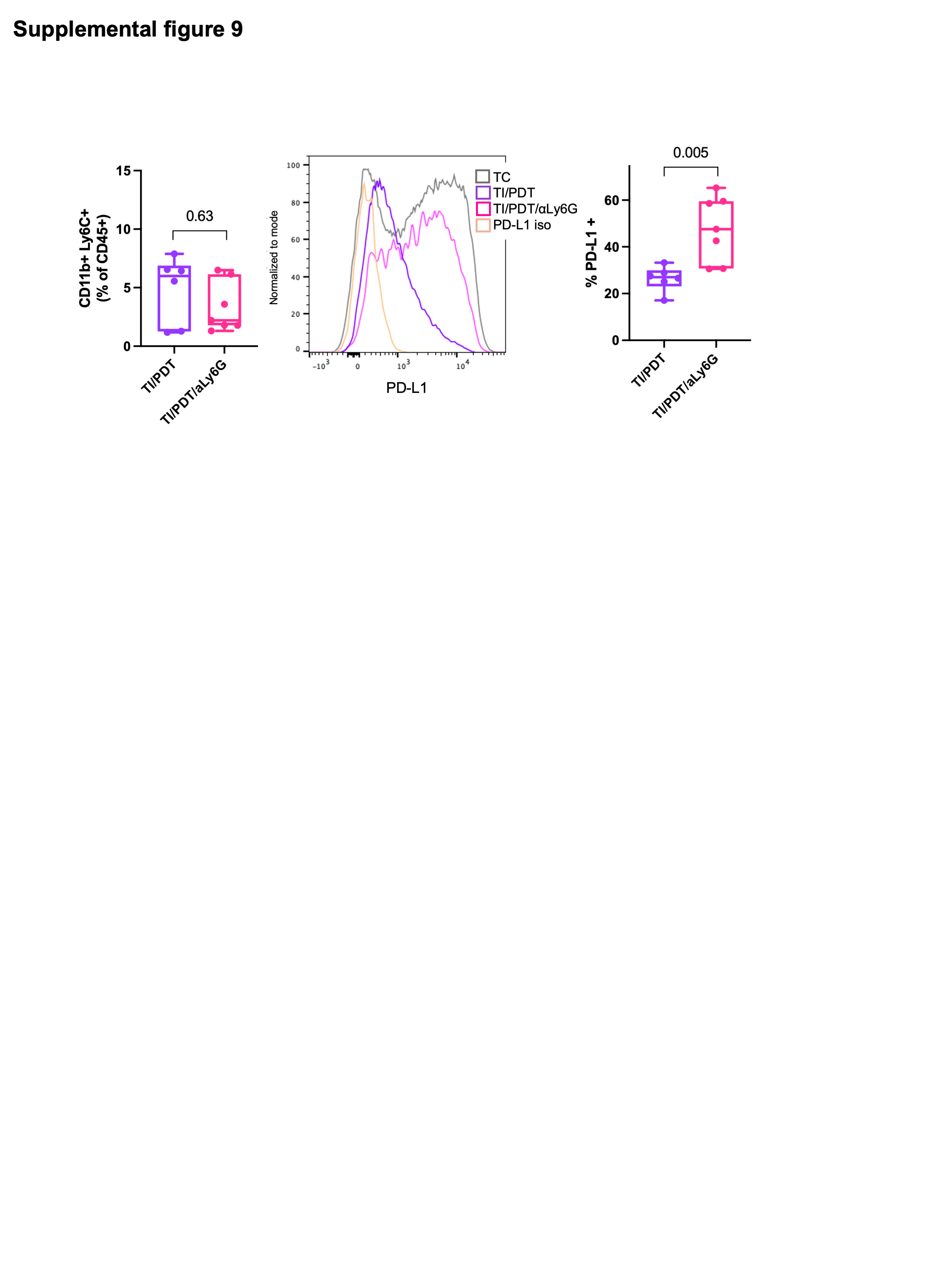

Supplement: Supplementary Figure 9 — Ly6G after TI/PDT depletion increases PD-L1 expression on Ly6C+ MDSCs [file crc-24-0571_supplementary_figure_9_suppsf9.png]
